# CEBPβ regulation of endogenous IGF-1 in adult sensory neurons can be mobilized to overcome diabetes-induced deficits in bioenergetics and axonal outgrowth

**DOI:** 10.1007/s00018-022-04201-9

**Published:** 2022-03-17

**Authors:** Mohamad-Reza Aghanoori, Prasoon Agarwal, Evan Gauvin, Raghu S. Nagalingam, Raiza Bonomo, Vinith Yathindranath, Darrell R. Smith, Yan Hai, Samantha Lee, Corinne G. Jolivalt, Nigel A. Calcutt, Meaghan J. Jones, Michael P. Czubryt, Donald W. Miller, Vernon W. Dolinsky, Virginie Mansuy-Aubert, Paul Fernyhough

**Affiliations:** 1 Division of Neurodegenerative Disorders, St. Boniface Hospital Albrechtsen Research Centre, Winnipeg, MB Canada; 2 Dept of Pharmacology and Therapeutics, University of Manitoba, Winnipeg, MB Canada; 3 Children’s Hospital Research Institute of Manitoba, University of Manitoba, Winnipeg, MB Canada; 4 Department of Physiology and Pathophysiology, Rady Faculty of Health Sciences, University of Manitoba, Winnipeg, MB Canada; 5 Institute of Cardiovascular Sciences, St. Boniface Hospital Albrechtsen Research Centre, Winnipeg, MB Canada; 6 Cellular and Molecular Department, Stritch School of Medicine, Loyola University Chicago, Chicago, USA; 7 Kleysen Institute for Advanced Medicine, University of Manitoba, Winnipeg, MB Canada; 8 Department of Biochemistry and Medical Genetics, Rady Faculty of Health Sciences, University of Manitoba, Winnipeg, MB Canada; 9 Department of Pathology, UCSD, La Jolla, San Diego, CA USA; 10 Present Address: School of Electrical Engineering and Computer Science, KTH Royal Institute of Technology, 10044 Stockholm, Sweden; 11 Dept of Medical Genetics, Cumming School of Medicine, University of Calgary, 3330 Hospital Drive NW, Calgary, AB T2N 4N2 Canada

**Keywords:** Dorsal root ganglia, Diabetic neuropathy, Mitochondria, NFAT1, Neurite outgrowth, Neurotrophic factor, IGF-1

## Abstract

Aberrant insulin-like growth factor 1 (IGF-1) signaling has been proposed as a contributing factor to the development of neurodegenerative disorders including diabetic neuropathy, and delivery of exogenous IGF-1 has been explored as a treatment for Alzheimer’s disease and amyotrophic lateral sclerosis. However, the role of autocrine/paracrine IGF-1 in neuroprotection has not been well established. We therefore used in vitro cell culture systems and animal models of diabetic neuropathy to characterize endogenous IGF-1 in sensory neurons and determine the factors regulating IGF-1 expression and/or affecting neuronal health. Single-cell RNA sequencing (scRNA-Seq) and in situ hybridization analyses revealed high expression of endogenous IGF-1 in non-peptidergic neurons and satellite glial cells (SGCs) of dorsal root ganglia (DRG). Brain cortex and DRG had higher IGF-1 gene expression than sciatic nerve. Bidirectional transport of IGF-1 along sensory nerves was observed. Despite no difference in IGF-1 receptor levels, IGF-1 gene expression was significantly (*P* < 0.05) reduced in liver and DRG from streptozotocin (STZ)-induced type 1 diabetic rats, Zucker diabetic fatty (ZDF) rats, mice on a high-fat/ high-sugar diet and *db/db* type 2 diabetic mice. Hyperglycemia suppressed IGF-1 gene expression in cultured DRG neurons and this was reversed by exogenous IGF-1 or the aldose reductase inhibitor sorbinil. Transcription factors, such as NFAT1 and CEBPβ, were also less enriched at the IGF-1 promoter in DRG from diabetic rats vs control rats. CEBPβ overexpression promoted neurite outgrowth and mitochondrial respiration, both of which were blunted by knocking down or blocking IGF-1. Suppression of endogenous IGF-1 in diabetes may contribute to neuropathy and its upregulation at the transcriptional level by CEBPβ can be a promising therapeutic approach.

**Supplementary Information:**

The online version contains supplementary material available at 10.1007/s00018-022-04201-9.

## Introduction

Insulin-like growth factor 1 (IGF-1) acts as a neurotrophic factor to promote neurite outgrowth from axotomized sensory [1–3], motor [4] and sympathetic [2, 5] neurons. IGF-1 and IGF-1 receptor (IGF1R) signaling are also implicated in survival, proliferation, migration and myelinating properties of Schwann cells [6–9]. Consequently, IGF-1 has been a therapeutic candidate against many disorders including Alzheimer’s disease, Fragile X syndrome, Rett syndrome, amyotrophic lateral sclerosis (ALS) and Parkinson’s disease [10]. For example, ALS patients receiving IGF-1 twice weekly for 40 weeks exhibited improved motor nerve function with no adverse side effects [11]. Four weeks of IGF-1 therapy to 12 female Rett syndrome patients improved mood stability, apnea and anxiety in a phase 1 clinical trial [12].

IGF-1 therapy also augments peripheral nerve regeneration and neuroprotection. For example, IGF-1 peptide improved nerve regeneration in both normal and streptozotocin (STZ)-induced type 1 diabetic rats after sciatic nerve crush [13–15]. Intrathecal delivery of IGF-1 reversed sensory and motor nerve conduction velocity deficits and prevented intra-epidermal nerve fiber (IENF) loss and axonal degeneration in the sural nerve of STZ-induced diabetic rats [16, 17]. Furthermore, overexpression of IGF-binding protein 5 (an intrinsic IGF-1 inhibitor) or depletion of IGF1R promoted a neurodegeneration phenotype in mice that resembled the nerve damage observed in humans with diabetic neuropathy [18]. IGF-1 activation of AMP-activated protein kinase (AMPK) in DRG sensory neurons may contribute to protection against neurodegeneration in type 1 diabetes [19]. Levels of systemic or background IGF-1 were markedly diminished in serum of humans and animal models of type 1 and type 2 diabetes [14, 20–22]. In addition, in the context of type 2 diabetes, studies in *ob*/*ob* mice revealed a loss of sensitivity to IGF-1 in DRG neurons [23]. Thus, suppression of IGF-1-mediated neurotrophic support and its signaling is proposed to contribute to neurodegeneration in diabetes [24].

Despite a plethora of evidence supporting the effects of systemic IGF-1 on cell phenotypes, including neuronal and non-neuronal survival and growth, little is known about *IGF-1* gene regulation and its function, especially with regard to the role of endogenous IGF-1 in sensory neurons. The *IGF-1* gene consists of six exons and five introns in humans (chromosome 12) and rodents that are differentially spliced to produce six different protein precursors from various transcript variants [25]. Several transcription factors including STAT5b, hepatocyte nuclear factors (HNF)-1, HNF-3, CCAAT/enhancer-binding protein (CEBP) α, β and δ have binding sites on the *IGF-1* gene promoter and regulate its transcription [26–30].

We hypothesized that impaired autocrine/paracrine actions of IGF-1 in DRG neurons contribute to progressive diabetic sensory neuropathy and therefore investigated the origin of endogenous IGF-1, its regulation at the transcriptional level and the mechanism of suppression of IGF-1 under hyperglycemic conditions in DRG-derived sensory neurons.

## Materials and methods

### Animals

Male Sprague Dawley rats (275–325 g) were used to model insulin deficient type 1 diabetes by delivery of a single intraperitoneal injection of 90 mg/kg STZ (Sigma, St Louis, MO, USA) to ablate pancreatic beta cells and were compared with age-matched control rats. Adult Zucker diabetic fatty (ZDF) rats and *db/db* mice were used as models of type 2 diabetes that exhibit insulin resistance, hyperinsulinemia and hyperglycemia, while C57BL/6 J mice fed a high-fat/high-sugar Western diet were used to model pre-diabetes with insulin resistance and hyperinsulinemia in the absence of overt hyperglycemia. For intervention studies, a subgroup of STZ-diabetic animals received 20 µg hIGF-1 peptide by intraperitoneal injection thrice weekly for 11 weeks, beginning 3 months after onset of diabetes. Fasting blood glucose concentration was monitored using an AlphaTRAK glucometer (Abbott Laboratories, Illinois, USA) to ensure that treatment did not affect hyperglycemia. At the study end, blood glucose, glycated hemoglobin (HbA1c Multi-test system, HealthCheck Systems, Brooklyn, NY, USA) and body weight were recorded before tissue collection. The neuropathy status of these animals has been described previously [19]. For Western diet studies, control C57BL/6 J mice were fed normal Chow (NC) (Teklad LM-485), while the experimental groups were fed Western Diet (WD) (TD88137, Teklad Diets; 42%kcal from fat, 34% sucrose by weight, and 0.2% cholesterol total—Envigo, Indiana, USA) for 14 weeks when glucose intolerance and neuropathy, as indicated by allodynia to von Frey filaments and heat hyperalgesia, had developed [31, 32]. In addition, mice used from this cohort displayed dermal and epidermal nerve fiber loss confirmed by PGP9.5 staining. Animal procedures were approved by the University of Manitoba Animal Care Committee and followed Canadian Council of Animal Care (CCAC) rules or followed National Institutes of Health guidelines with approval by the local Institutional Animal Care and Use Committees at Loyola University Chicago or the University of California San Diego.

### Adult DRG sensory neuron and HEK293 cell culture

DRG neurons were isolated from adult male Sprague Dawley (300-350 g) rats, dissociated and cultured using previously described methods [33]. Briefly, neurons were cultured in glucose-free Hams F12 media supplemented with Bottenstein’s N2 without insulin (Sigma, St Louis, MO, USA). DRG neurons from control rats were cultured in the presence of 5 mM D-glucose and DRG neurons derived from STZ-induced diabetic rats with 25 mM D-glucose. No neurotrophins or insulin was added to any DRG cultures unless otherwise indicated. The HEK293 (ATCC CRL-1573, Virginia, USA) cell line was cultured in DMEM/F12 (1:1) media containing 10 mM glucose and 10% FBS. Sorbinil, a selective inhibitor of aldose reductase was purchased from a commercial supplier (Sigma, St Louis, MO, USA). For more details, see Supplementary Information.

### Preparation of lipid nanoparticle (LNP) formulations for siRNA and pDNA applications

Lipid nanoparticle–siRNA (LNP–siRNA) was used to knock down endogenous IGF-1 in DRG neurons. For this purpose, a mixture of siRNA-29, 5′-GCUGAAGCCUACAAAGUCAtt-3′ (siRNA ID: s127929, Thermo Scientific, Pittsburgh, PA, USA) and siRNA-31, 5′-GAAGUACACUUGAAGAACAtt-3′ (siRNA ID: s127931, Thermo Scientific, Pittsburgh, PA, USA) specific to rat IGF-1, or a scrambled siRNA (Cat #:4635, Thermo Scientific, Pittsburgh, PA, USA) as a negative control was used. All LNP–siRNA, LNP–GFP (AG13105-CH, Sino Biological Inc., Beijing, China), LNP–IGF-1 (HG29626-NH, Sino Biological Inc., Beijing, China) and LNP–CEBPβ (Addgene, USA; plasmid # 12557) overexpressing plasmids were formulated using Neuro9 (Precision NanoSystems, Vancouver, BC, Canada) transfection kit following microfluidics mixing method using the NanoAssemblr Benchtop instrument (Precision NanoSystems, Vancouver, BC, Canada) following supplied protocol. See Supplementary Information for more details.

### Quantitative Western blotting

Rat DRG neurons/tissues were harvested from culture or isolated intact from adult rats or mice and then homogenized in ice-cold RIPA buffer for Western blotting. See Supplementary Information for more details.

### ELISA assay

Homogenized tissues or collected media from DRG cultures were assayed using the Mouse/Rat IGF-1 Quantikine ELISA kit (R&D Systems, Minnesota, USA) according to the kit instructions. All media collected from cultures were tested for secreted IGF-1.

### RNA isolation, cDNA library construction and illumina sequencing

See Supplementary Information for more details [34].

### Real-time PCR

RNA was extracted from cultured neurons or frozen tissue samples using TRIzol® Reagent (Invitrogen, California, USA). Complementary DNA (cDNA) was synthesized from RNA samples using the iScript™ gDNA Clear cDNA Synthesis Kit (Bio-Rad, CA, USA) according to the manufacturer's instructions. See Supplementary Information for more details.

### Chromatin immunoprecipitation (ChIP) assay

DRG were dissected from rats, weighed and underwent ChIP analysis assay using the ChromaFlash™ High-Sensitivity ChIP Kit (Catalog # P-2027, Epigentek, Farmingdale, NY, USA). See Supplementary Information for more details. Fold enrichment was calculated using the formula FE = 2^(IgG CT − sample CT)^ to identify binding sites for NFAT1 or CEBPβ on the IGF-1 promoter.

### RNA FISH

We designed 28 oligonucleotide probes, 18–26 bp in length, spanning the whole rat IGF-1 mRNA. Probes were fluorescently labeled with Quasar 570 (Stellaris RNA FISH, Biosearch Technologies, Petaluma, CA, USA) for imaging using a Carl Zeiss Axioscope-2 upright fluorescence microscope equipped with AxioVision3 software. To visualize IGF-1 mRNA in the cultured neurons or DRG tissue sections, we followed the protocol for adherent cells and frozen tissues (Stellaris RNA FISH, Biosearch Technologies, Petaluma, CA, USA). See Supplementary Information for more details. Twenty images per group with a magnification of 63X were captured. A culture/section group without any probe or treated with RNase A (50 µg/mL) for 30 min at 37 °C, prior to the hybridization step was used as a negative control.

### Northern blotting

RNA was extracted from DRG, sciatic nerve and brain tissues from rat using TRIzol® Reagent (Invitrogen, California, USA). A modified protocol from the Hackett lab (https://cbs.umn.edu/hackett-lab/protocols/northern-blotting) for Northern blotting and hybridization was used. See Supplementary Information for more details. Two micrograms (2 µg) of total RNA was used and run on a 1.2% agarose gel to visualize 5S, 18S and 28S rRNA from each tissue sample for normalization of Real-Time PCR and Northern blotting results.

### Site-directed mutagenesis on IGF-1 promoter and luciferase-reporter assay

Rat IGF-1 gene promoter (about 1.2 kb upstream to exon 1) was amplified using Q5 high-fidelity DNA polymerase (Cat#: M0491G, New England Biolabs, Massachusetts, USA) with primers having HindIII and XhoI recognition sites at their 5’ ends. PCR product was treated with these restriction enzymes (New England Biolabs, Massachusetts, USA), ligated into pGL4.10 [luc2] (Promega, Wisconsin, USA) and transformed into DH5a cells according to the manufacturer’s instructions (New England Biolabs, Massachusetts, USA). Q5® Site-Directed Mutagenesis Kit (New England Biolabs, Massachusetts, USA) and 4 pairs of primers were used to make mutated binding sites for NFAT1 or CEBPβ transcription factors in the IGF-1 promoter part of the construct. Plasmids were purified from single colonies, sequenced for validation, and co-transfected with pcDNA 3.1( +) NFAT1 (subcloned from pENTR11 WT NFAT1, Addgene plasmid # 11791; USA) or pcDNA 3.1(-) mouse CEBPβ (LAP) (a gift from Peter Johnson, Addgene plasmid # 12557; USA) into HEK293 cell line. IGF-1 promoter-driven luciferase activity was measured and recorded as bioluminescence unit using Glomax-multi detection system (Promega, Wisconsin, USA). Emitted bioluminescence was normalized to Renilla emissions and plotted.

### Mitochondrial respiration and glycolysis assay in cultured neurons and HEK293 cells

Mitochondrial oxygen consumption rate (OCR) was measured in live sensory neurons and HEK293 cells using the XF24 analyzer (Seahorse Biosciences, Billerica, MA, USA). For the glycolysis test, glucose (10 mM), oligomycin (1 µM) and 2-deoxy-glucose (2DG: a glucose analog) (50 mM) were sequentially injected to achieve extracellular acidification rate (ECAR). See Supplementary Information for more details.

### Immunocytochemistry

DRG neurons were cultured on glass coverslips, transfected/treated, and underwent neurite outgrowth analysis after a specified time. See Supplementary Information for more details.

### Pyrosequencing

DNA was extracted from DRG of diabetic and control rats and underwent bisulfite conversion per manufacturer’s instructions (Cat#D5020, Zymo Research, USA). Biotinylated primers and the pyrosequencing assays were designed using PyroMark Assay Design 2.0 (Qiagen, Inc.) software to cover 7 CpG sites on IGF-1 promoter. PCR and pyrosequencing performed as previously described [35]. Sequencing primers were then added for pyrosequencing per manufacturer’s instructions (Pyromark™ Q96 MD Pyrosequencer, Qiagen, Inc.). See Supplementary Information for more details.

### Single-cell and whole DRG RNA sequencing data acquisition and analysis

Normalized expression level (RPM) data from single-cell RNA-Seq (scRNA-Seq) study of mouse DRG were obtained from publicly available datasets (GSE59739 in GEO database) which were previously deposited by Usoskin et al. [36]. The expression levels of markers of each DRG subpopulation together with *Igf1* and *Igf1r* expression levels were extracted and analyzed. The markers for each DRG cell population used were as follows: NF cluster (myelinated neurons): neuro-filament heavy chain (NEFH), parvalbumin (PVALB) and BIII-tubulin (TUBB3), PEP cluster (peptidergic nociceptors): substance P (TAC1), TrkA (NTRK1), calcitonin gene-related peptide (CALCA) and BIII-tubulin (TUBB3), NP cluster (non-peptidergic nociceptors): purinergic receptor P2X 3 (P2RX3) and BIII-tubulin (TUBB3), TH cluster (Type C low-threshold mechanoreceptors): tyrosine hydroxylase (TH) and BIII-tubulin (TUBB3), SC cluster (Schwann cells): P75 receptor and myelin basic protein (MBP), SGC cluster (Satellite glial cells): glycogen synthase (GS) and S100 calcium-binding protein B (S100B). Relative IGF-1 and IGF1R expression levels were plotted to compare the mean and distribution in each DRG subgroup.

### Statistical analysis

Data were analyzed using two-tailed Student’s *t *tests or one-way ANOVA followed by Tukey’s or Dunnett’s post hoc tests, as appropriate and indicated (GraphPad Prism 7, GraphPad Software). A *P* value < 0.05 was considered significant.

## Results

### Endogenous IGF-1 is expressed at high levels in specific subpopulations of DRG neurons, and is transported bi-directionally along axons in vivo

**Fig. 1:**
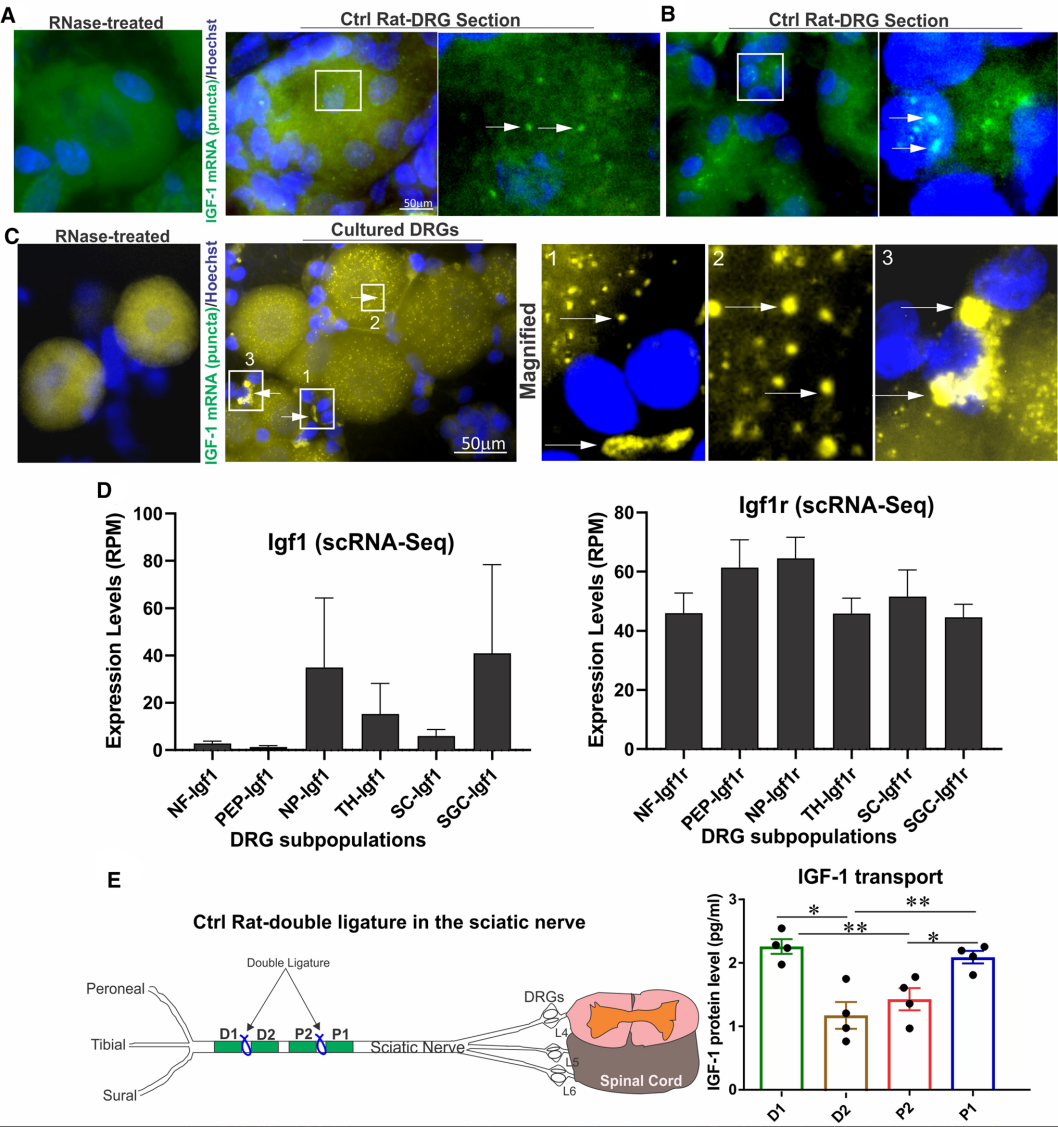
Cellular localization and axonal transport of endogenous IGF-1 in DRG. **A**, **B** DRG tissue sections or **C** cultured DRG neurons from control rats underwent RNA FISH assay for IGF-1 mRNA detection and localization. A punctate pattern of IGF-1 mRNA is evident in neurons and a limited number of associated glia. As a control, tissue sections and cells were exposed to RNase enzyme before hybridizing with IGF-1 probes. Images are magnified for clarification. As a control, tissue sections and cells were exposed to RNase enzyme before hybridizing with IGF-1 probes. IGF-1 mRNA punctate labeling is shown in **A** neurons and **B** glia in DRG tissue sections. In **C**, cultured DRG neurons and associated glia showed punctate staining for IGF-1. Boxes 1–3 are magnified parts of the figure shown for clarity. Arrows show glial and neuronal IGF-1 in box1, neuronal IGF-1 in box2 and glial IGF-1 in box3. In **D**, publicly available scRNA-Seq normalized data (RPM) from 240 to 480 cells was analyzed and relative transcript levels of Igf1 and Igf1r were plotted for each DRG subpopulation: NF cluster (myelinated neurons), PEP cluster (peptidergic nociceptors), NP cluster (non-peptidergic nociceptors), TH cluster (type-C low-threshold mechanoreceptors), SC cluster (Schwann cells) and SGC cluster (satellite glial cells). In **E**, a 12-h double ligature (1 cm width) experiment was carried out on rat sciatic nerve with four regions proximal 1 (P1), P2, distal 1 (D1) and D2 analyzed for IGF-1 levels. Data are mean ± SEM of *N* = 3–4. *p < 0.05 or **p < 0.01; analyzed by one-way ANOVA with Tukey’s *post hoc* test

To investigate the expression of endogenous IGF-1 by DRG neurons, we conducted RNA FISH assay to detect IGF-1 mRNA using specific fluorescent probes within DRG neurons and associated satellite glial cells (SGCs) and Schwann cells (SCs). Endogenous IGF-1 mRNA was detected as puncta in liver (Supplemental Fig. 1A) and DRG from control rats (Fig. 1A, B), but not when tissue sections and cells were exposed to RNase enzyme before hybridizing with IGF-1 probes. In cultured DRG neurons, endogenous IGF-1 mRNA was detected predominantly in neurons and a subset of glial cells (Fig. 1C). Schwann cells provide the majority of nuclei in peripheral nerves, along with vascular endothelial cells and perineurial cells. Using quantitative Northern blotting, we found that the hierarchy of IGF-1 mRNA expression levels was brain cortex > DRG > sciatic nerve (Supplemental Figure 1B).

The RNA integrity of brain cortex, DRG and sciatic nerve tissue was assessed with 5S rRNA, 18S rRNA and 28S rRNA bands used for normalization (Supplemental Figure 1C). IGF-1 mRNA detected by Real-Time PCR, was significantly (*P* < 0.001) higher in DRG tissue versus sciatic nerve (Supplemental Figure 1D). The highest level of IGF-1 mRNA was detected in the brain cortex compared to DRG tissue and sciatic nerve (Supplemental Figure 1E).

Since there was no single marker to distinguish subpopulations of cells in DRG tissue, it was not possible to determine which cells specifically expressed and responded to IGF-1 using FISH. We therefore determined which subpopulation of DRG neurons had the highest and lowest IGF-1 transcript levels by analyzing available scRNA-Seq data derived from mouse DRG and using a published clustering system [36]. Trends to higher levels of IGF-1 mRNA were seen in non-peptidergic nociceptors, mechanoreceptors and satellite cells compared to Schwann cells, peptidergic nociceptors and myelinated neurons expressing neuro-filament heavy chain (Fig. 1D). IGF-1R transcript was less variable across DRG neuron subpopulations (Fig. 1D). Expression levels of IGF-1 and IGF-1R were comparable to IGF-2 and IGF-2R in each category of cell types in DRG from mice (data not shown).

To determine if endogenous expression of IGF-1 mRNA by DRG neurons was associated with axonal transport of IGF-1 protein, we applied a double ligature to the sciatic nerve in adult rats for 12 h (Fig. 1E). Higher levels of endogenous IGF-1 collecting at proximal (P1) and distal (D1) portions of the nerve compared with internal segments (P2 and D2) confirmed anterograde and retrograde axonal transport of IGF-1(Fig. 1E).

### The level of IGF-1 is reduced in the liver and DRG tissue from type 1 and type 2 diabetic rodents and is restored by exogenous hIGF-1 treatment

**Fig. 2:**
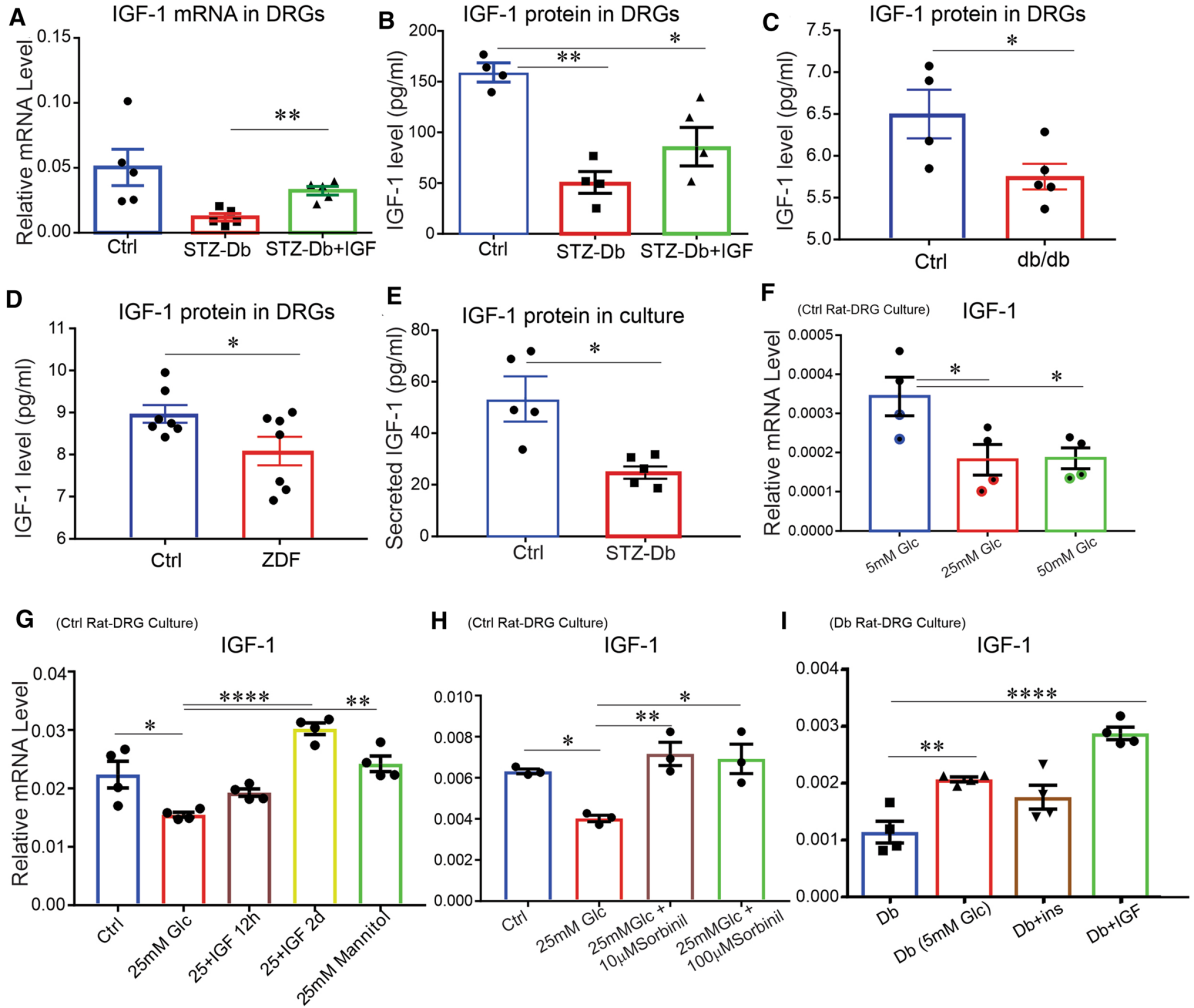
The level of endogenous IGF-1 was reduced in DRG tissue from type 1 and type 2 diabetic rodents, in part, via hyperglycemia-activated polyol pathway activity and restored by exogenous hIGF-1. In **A**–**D** DRG tissues from control (Ctrl), hIGF-1-treated (STZ-Db + hIGF-1), untreated STZ-diabetic (STZ-Db), Zucker diabetic fatty (ZDF) rats and *db/db* mice were homogenized and underwent **A** qRT-PCR or **B**–**D** ELISA for IGF-1 detection. In **E**, DRG neurons derived from Ctrl and STZ-Db rats were cultured and media was collected after 2 days to measure secreted IGF-1 protein. DRG neurons derived from control (**F**, **G**, **H**) or diabetic (**I**) rats were cultured in the presence of 25 mM glucose with/without insulin or hIGF-1 treatment or in the presence of 5 mM glucose. RNA was extracted and utilized for real-time PCR assay. In **G**, 25 mM mannitol was used to control for osmotic pressure compared with 25 mM D-glucose. In **H**, sorbinil (10 and 100 µM), an aldose reductase inhibitor (ARI), was applied. Data are mean ± SEM of *N* = 3–6; *p < 0.05 or **p < 0.01 or ****p < 0.0001; analyzed by Student’s *t *test or one-way ANOVA with Dunnett’s or Tukey’s *post hoc* test

IGF-1 protein levels in liver from hIGF-1-treated and untreated STZ-diabetic rats were significantly (*P* < 0.01) lower than those in control rats (Supplemental Figure 1F). DRG also expressed mRNA for IGF-1, and this endogenous expression showed a trend of reduction in the diabetic state which was significantly up-regulated by exogenous hIGF-1 treatment (Fig. 2A). Similarly, there was a significant decrease in endogenous IGF-1 protein level in DRG from STZ-diabetic rats, with a trend to higher protein levels in the hIGF-1 treated group (Fig. 2B). In contrast, the level of IGF-1 receptor β (IGF-1Rβ) protein was not different between groups (data not shown). In agreement with data from STZ-diabetic rats, the level of endogenous IGF-1 protein was significantly (*P* < 0.05) lower in DRG from two models of type 2 diabetes, *db*/*db* mice (Fig. 2C) and ZDF rats (Fig. 2D), compared to their control counterparts. Furthermore, pre-diabetic mice maintained on a high-fat high-sugar Western diet for 14 weeks exhibiting diabetic neuropathy displayed a 3.7-fold decrease in IGF-1 transcripts in DRG (*q *value = 0.000019) (Supplemental Figure 1G). Transcript levels of IGF-1R did not change over the 14-week Western diet protocol.

### Endogenous IGF-1 gene expression in DRG neuron culture is suppressed by polyol pathway and restored by exogenous hIGF-1

To further investigate the mechanism through which IGF-1 gene expression is regulated under diabetic conditions, we measured IGF-1 mRNA in different cell culture groups. IGF-1 transcript variants 3 and 4 were expressed at higher levels compared with transcript variants 1 and 2 in DRG from normal rats (data not shown), so we focused on IGF-1 transcript variants 3 and 4 for further studies. We collected the conditioned media from DRG cultures derived from control or STZ-diabetic rats and measured endogenous IGF-1 production and secretion using an IGF-1 ELISA assay. There was a significant reduction (*P* < 0.05) of IGF-1 protein secretion from DRG neuron cultures derived from STZ-diabetic rats when compared to those from control rats (Fig. 2E). A significant (*P* < 0.05) suppression of endogenous IGF-1 gene expression was observed when cultured DRG neurons derived from control rats were exposed to 25 mM or 50 mM D-glucose for 2 days (Fig. 2F).

Exogenous hIGF-1 treatment for 2 days, but not 12 h, restored the level of endogenous IGF-1 mRNA in DRGs derived from control rats that were exposed to 25 mM D-glucose (Fig. 2G). Mannitol had no effect, indicating that any osmotic effect of high glucose concentrations was not responsible for the inhibition of IGF expression. Two concentrations (10 and 100 µM) of sorbinil, an aldose reductase inhibitor (ARI), prevented the suppression of IGF-1 gene expression in cultured DRG neurons from control rats under hyperglycemic (25 mM glucose) conditions suggesting polyol pathway activity was inducing an inhibitory effect on IGF-1 at the transcriptional or post-transcriptional level (Fig. 2H). In DRG derived from STZ-diabetic rats and grown in the presence of 25 mM glucose, exogenous hIGF-1 (10 nM) treatment but not insulin (10 nM) increased (*P* < 0.0001) the level of endogenous IGF-1 (Fig. 2I). Exposure of diabetic neurons to 5 mM D-glucose for 1 day also induced significantly higher levels of IGF-1 mRNA compared with the 25 mM D-glucose group, revealing that the inhibitory effect of hyperglycemia on endogenous IGF-1 gene expression could be relieved by transition to normoglycemia (Fig. 2I).

### Manipulation of endogenous IGF-1 signaling modulates neurite outgrowth and bioenergetics in cultured DRG neurons

**Fig. 3:**
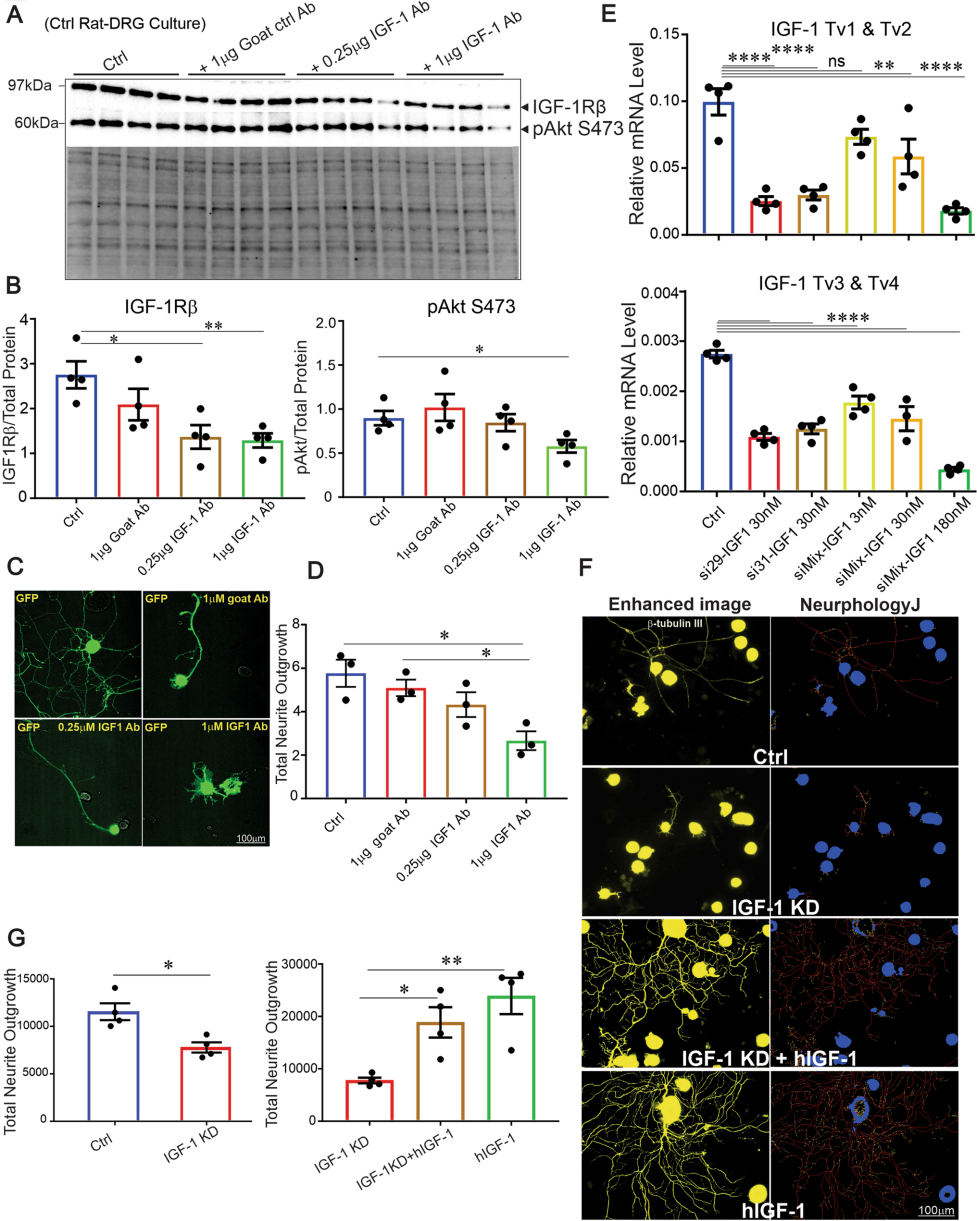
IGF-1 neutralizing antibody or IGF-1 targeting siRNAs reduced IGF-1Rβ and Akt phosphorylation and diminish neurite outgrowth. DRG neurons from control rats were cultured, treated with different doses of IGF-1 neutralizing antibody and underwent **A**, **B** Western blotting for Akt phosphorylation and IGF-1Rβ or **C**, **D** neurite outgrowth measurement. In **A**, **B**, total protein bands were used for normalization. In **C**, **D**, normal goat IgG was used as a control antibody. In **E**–**G**, DRG neurons from control rats were cultured, treated with different doses of two IGF-1 targeting siRNAs and underwent **E** real-time PCR assay for IGF-1 or **F**, **G** neurite outgrowth measurement. In **E**, four transcript variants (a pair of primers designed for the detection of two variants 1 and 2 or 3 and 4) of IGF-1 that produce protein were analyzed. In **F**, **G**, exogenous hIGF-1 was added alone or along with IGF-1 knock-down as control groups. Data are mean ± SEM of *N* = 4; *p < 0.05 or **p < 0.01 or ****p < 0.0001; analyzed by Student’s *t *test or one-way ANOVA with Dunnett’s *post hoc* test

DRG neurons derived from age-matched control rats were cultured for 2 days in the presence of 1 µg IGF-1 neutralizing antibody, which resulted in lower phosphorylated Akt (at S473) and IGF-1Rβ compared to DRG neurons grown under control conditions (Fig. 3A, B). This effect was associated with concomitant inhibition of neurite outgrowth (Fig. 3C, D). To confirm the significance of endogenous IGF-1 secretion for supporting neurite outgrowth, we used two specific IGF-1 siRNAs encapsulated in lipid nanoparticles (LNPs) for higher knock-down efficiency and lower toxicity (Fig. 3E). These effects were dose-dependent; with 180 nM mixed siRNAs giving the highest level of IGF-1 knock-down (approximately 85% reduction). The 180 nM siRNA dose reduced background neurite outgrowth in DRG neuron cultures derived from control rats (*P* < 0.05) (Fig. 3F, G). This suppressed neurite outgrowth was significantly overcome by co-treatment with exogenous hIGF-1 (*P* < 0.05). Similar enhancement of neurite outgrowth was also observed when hIGF-1 was used as the only treatment (Fig. 3F, G). In a complementary experiment, IGF-1 neutralizing antibody or siRNA both suppressed the upregulation of mitochondrial respiration and neurite outgrowth produced by a cocktail of growth factors (NGF, GDNF and NT-3) in cultured DRG neurons from diabetic rats (Supplemental Figure 2A, B).

**Fig. 4:**
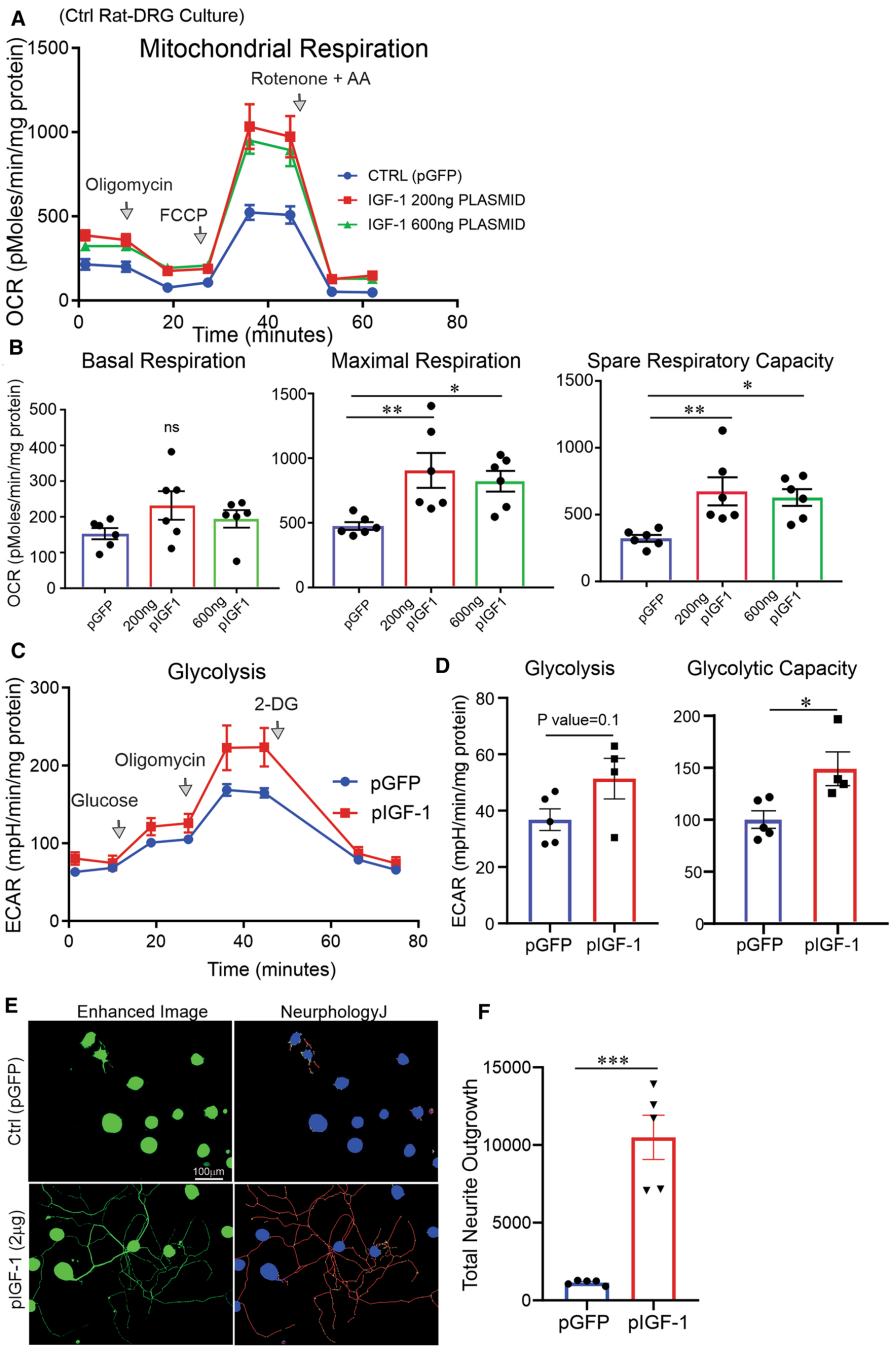
IGF-1-overexpressing plasmid enhanced glycolysis, mitochondrial respiration, and neurite outgrowth. DRG neurons from control rats were cultured, transfected with different doses of hIGF-1 (transcript variant 4)-overexpressing plasmid and 200 ng control GFP plasmid (ctrl). In **A**, **B**, mitochondrial OCR was measured in live neurons after 6 h. In **C**–**F**, glycolysis parameters and total neurite outgrowth were calculated. Total protein in mg was used to normalize OCR and ECAR data. Data are mean ± SEM of *N* = 4–6; *p < 0.05 or **p < 0.01 or ***p < 0.001; analyzed by Student’s *t *test or one-way ANOVA with Dunnett’s *post hoc* test

Overexpression of IGF-1 via treatment with pIGF-1–LNP (200 ng or 600 ng) in cultured DRG neurons increased mitochondrial maximal respiration, spare respiratory capacity, as well as glycolysis and glycolysis capacity, 36 h following transfection (Fig. 4A–D). The transfections with pIGF-1–LNP resulted in a significant (*P* < 0.001) increase in neurite outgrowth in DRG neurons derived from control rats (Fig. 4E, F). HEK293 cells overexpressing IGF-1 also exhibited augmented mitochondrial function (Supplemental Figure 3A–D).

### Binding of NFAT1 and CEBPβ transcription factors to the IGF-1 promoter is diminished in DRG from diabetic rats

**Fig.5:**
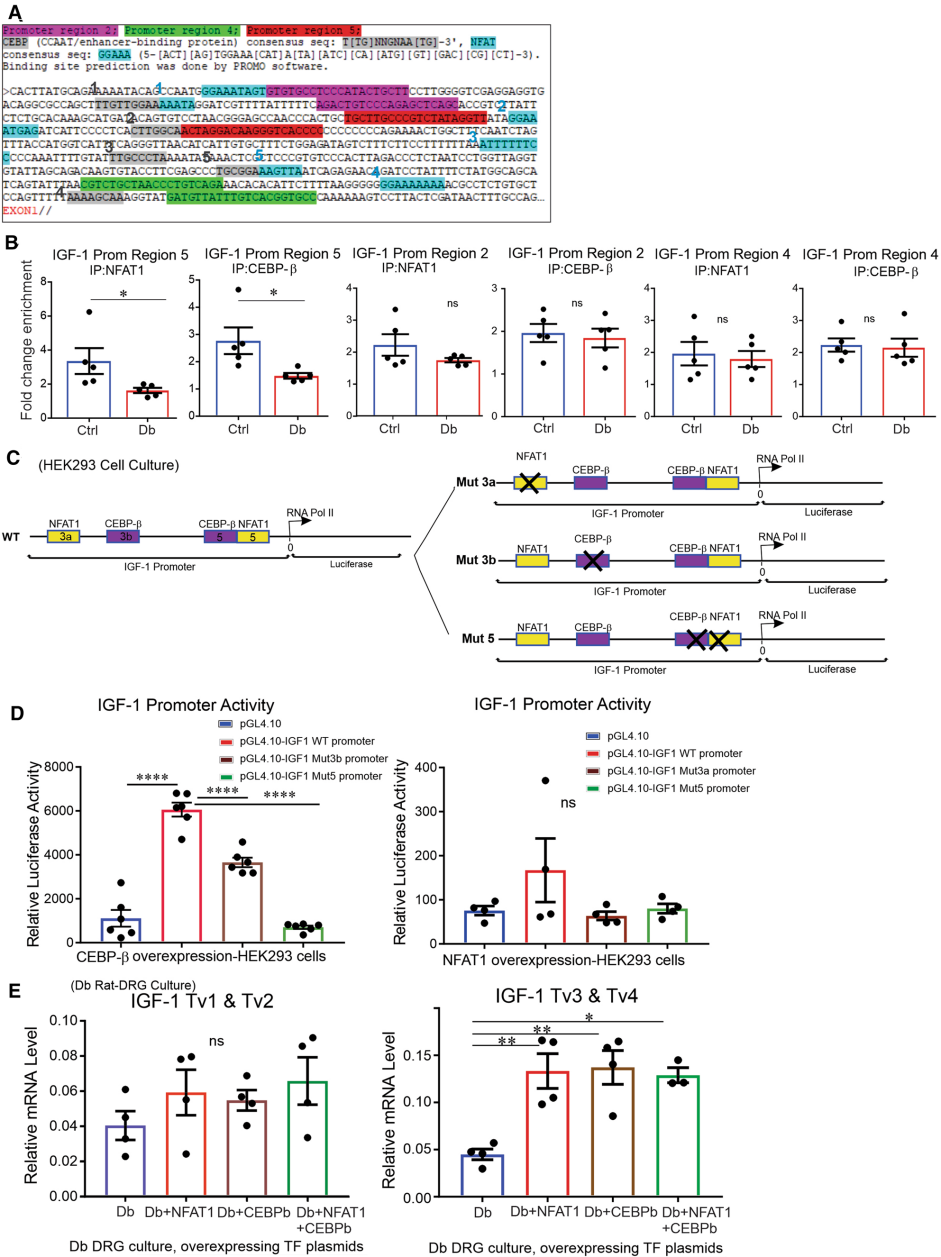
NFAT1 and CEBPβ transcription factors activated IGF-1 gene expression and were less enriched at the Igf1 promoter in DRG tissue derived from diabetic rats. About **A** 1.2 kb of IGF-1 gene promoter region was chosen to design ChIP assay. Five regions were used for amplification and each pair of primers was designed to include one transcription factor-binding site. In **B**, DRG tissues derived from adult control and STZ-diabetic rats underwent ChIP assay using NFAT1 and CEBPβ antibodies for pull down followed by IGF-1 promoter region (five regions in total stats for three are shown) amplification using ChIP-qRT-PCR analysis. In **C**, diagram of the mutated transcription factor (NFAT1 and CEBPβ) binding sites on promoter region of Igf1 gene is given. A total of 4 binding sites (3a for NFAT1-binding site 3, 3b for CEBPβ-binding site 3, 5 for NFAT1- and CEBPβ-binding site 5) from (**A**) were mutated. In **D**, luciferase activity of the mutated and wild type IGF-1 promoter was measured in HEK293 cells. In **E**, DRG neurons from STZ-diabetic (Db) rats were transfected with NFAT1, CEBPβ or both, and different transcript variants (Tv1,2,3,4) of IGF-1 were measured using qRT-PCR. Data are mean ± SEM of *N* = 4–5 animals or *N* = 4–6 culture groups; *p < 0.05 or **p < 0.01 or ****p < 0.0001; analyzed by Student’s *t *test or one-way ANOVA with Tukey’s *post hoc* test

Bioinformatic screening of the IGF-1 promoter (two defined promoters for all 4 transcript variants of the *Igf1* gene) revealed several transcription factor-binding sites including those specific to NFAT1 and CEBPβ transcription factors with highest hits, which were evaluated (Supplemental Figure 4A). Of note, chromatin fragmentation was optimized before the ChIP assay (Supplemental Figure 4B). Five amplicons (five promoter regions) covering 1.2 kb upstream of rat *Igf1* gene were considered for Real-Time PCR, and the specificity of the corresponding primers was validated on an agarose gel (Supplemental Figures 4C, 5A). One out of five regions of the IGF-1 promoter (promoter region 5: close to transcription start site) was enriched for NFAT1 and CEBPβ binding in DRG from control rats when compared to DRG from STZ-diabetic rats (*P* < 0.05) (Fig. 5A, B). However, the levels of both transcription factors (Supplemental Figure 5A, B) and IGF-1 promoter DNA methylation (Supplemental Figure 5C) remained unchanged in DRG derived from STZ-diabetic rats compared with control rats, highlighting the importance of endogenous transcription factor activity. Furthermore, luciferase assay analysis revealed that abolishing one specific binding site (region 5 mutated) for CEBPβ on the IGF-1 promoter could completely suppress the luciferase activity induced by CEBPβ overexpression in HEK293 cells, although other mutated binding sites for NFAT1 and CEBPβ showed a similar trend of suppression (Fig. 5C, D). Overexpression of both transcription factors enhanced (*P* < 0.01) endogenous IGF-1 transcript variants 3 and 4 in DRG neurons cultured from STZ-diabetic rats (Fig. 5E). Efficient overexpression of NFAT1, CEBP-β, GFP and IGF-1 genes in HEK293 cells was confirmed prior to these assays (Supplemental Figure 6A–F).

### Transcription factor CEBPβ enhances mitochondrial respiration and neurite outgrowth through upregulation of IGF-1 gene expression

To explore the downstream effects of the two transcription factors on bioenergetics, we measured mitochondrial respiration and glycolysis in DRG neurons transfected with NFAT1- or CEBPβ-overexpressing plasmids. First, we showed that the transfection efficiency of pGFP–LNP in cultured DRG neurons was satisfactory, with the highest transfection efficiency at the dose of 300 ng pGFP–LNP complex (data not shown). We then developed an encapsulation strategy to transfect DRG neurons with the pIGF-1–LNP or pCEBPβ–LNP to achieve the highest efficiency.

**Fig. 6:**
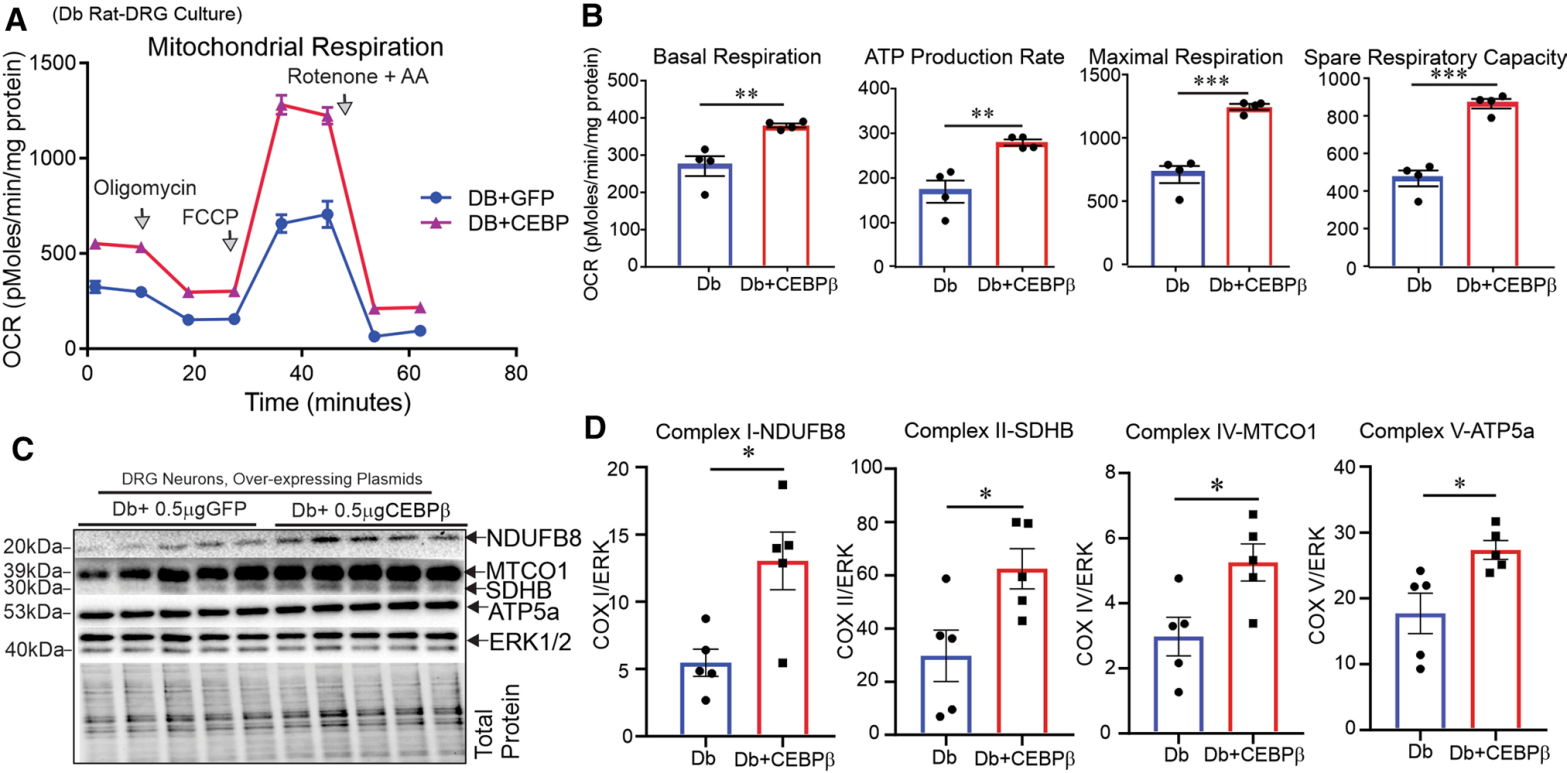
CEBPβ overexpression elevated mitochondrial ETS protein levels and mitochondrial respiration. DRG neurons from **A**–**D** STZ-diabetic rats were cultured, transfected with CEBPβ-overexpressing plasmid or control GFP plasmid. In **A**, **B**, mitochondrial OCR was measured in live neurons after 36 h. In **C**, **D**, mitochondrial ETS proteins were measured using Western blotting. Total protein band was used to normalize Western blot data. Data are mean ± SEM of *N* = 4–6; *p < 0.05 or **p < 0.01 or ***p < 0.001; analyzed by Student’s *t *test

**Fig. 7:**
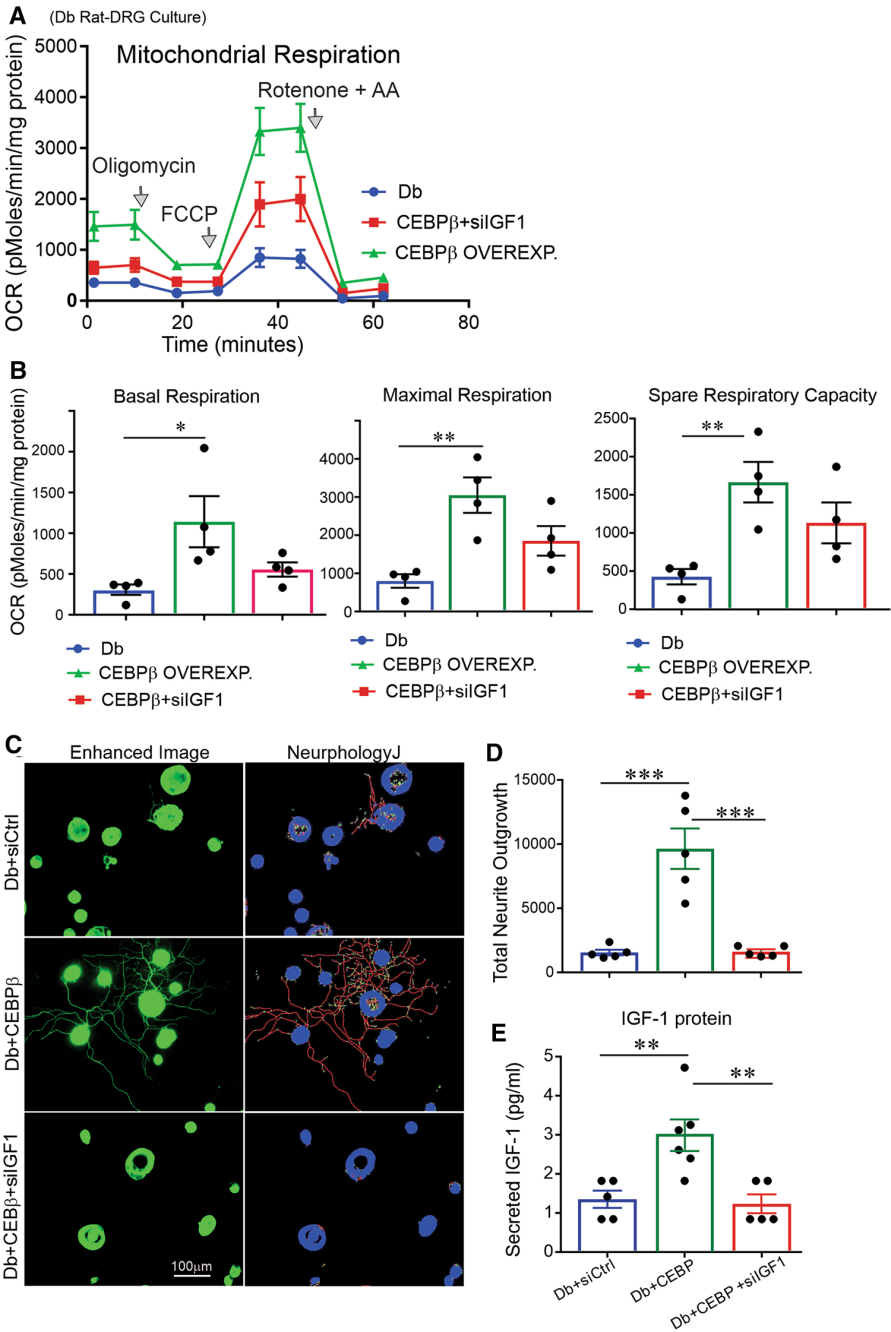
IGF-1 knock-down suppressed CEBPβ upregulation of mitochondrial function and neurite outgrowth. DRG neurons from STZ-diabetic (Db) rats were cultured, transfected with pCEBPβ–LNP, pGFP–LNP or siIGF1–LNP, and underwent **A**, **B** mitochondrial respiration assay or **C**, **D** neurite outgrowth measurement. In **A**, **B**, total protein in mg was used to normalize OCR data. In **E**, ELISA assay was used to measure IGF-1 levels in the same condition as in **C**. Data are mean ± SEM of *N* = 3–5; *p < 0.05 or **p < 0.01 or ***p < 0.001; analyzed by one-way ANOVA with Tukey’s *post hoc* test

Mitochondrial ATP production rate, basal respiration, maximal respiration and spare respiratory capacity were significantly increased when cultured DRG neurons from diabetic rats were transfected with pCEBPβ–LNP (Fig. 6A, B). In addition, CEBPβ overexpression increased expression of mitochondrial electron transport system (ETS) proteins NDUFB8, SDHB, MTCO1 and ATP5a in rat DRG neurons (Fig. 6C, D) and elevated mitochondrial respiration in HEK293 cells, despite no significant effect on glycolysis (Supplemental Figure 7A–D). Transfection with encapsulated siIGF-1 (treatment with siRNA to IGF-1) abolished the CEBPβ-dependent enhancement of mitochondrial respiration (Fig. 7A, B) indicating involvement of endogenous IGF-1 in CEBPβ upregulation of mitochondrial function. When neurons were transfected with pCEBPβ–LNP, they produced higher levels of secreted IGF-1 and promoted robust neurite outgrowth in DRG neurons. Transfection with encapsulated siIGF-1 for 1 h prior to CEBPβ transfection inhibited the effect of CEBPβ on neurite outgrowth and IGF-1 protein levels (Fig. 7C, E).

A similar strategy was employed to assess the effect of the NFAT1–IGF1 axis on bioenergetics in HEK293 cells and primary cultures of rat DRG. NFAT1 up-regulated glycolysis and glycolytic capacity in DRG neurons from control rats and in HEK293 cells transfected with NFAT1-overexpressing plasmid. Basal and maximal respiration and ATP production rate were also upregulated in HEK293 cells following NFAT1 overexpression (Supplemental Figure 8A–F). In contrast, NFAT1 overexpression only upregulated ATP5a in DRG neurons derived from control rats (Supplemental Figure 9A, B). Inhibition of IGF-1 transcription partially blunted the effect of NFAT1 overexpression on mitochondrial respiration parameters (Supplemental Figure 9C–D).

## Discussion

We have demonstrated that endogenous production of IGF-1, detected at both the mRNA and protein levels in DRG tissue and culture, is suppressed under hyperglycemic conditions via effectors mediated, in part, by the polyol pathway. We further demonstrate that exogenous hIGF-1 exerted positive feedback on endogenous IGF-1 transcript levels under hyperglycemic conditions. Endogenous IGF-1 was expressed at a higher level in the DRG compared with sciatic nerve, while within the DRG higher mRNA expression was detected in two specific subtypes of sensory neuron and in satellite cells. IGF-1 exhibited anterograde transport along axons in vivo. In addition, we provide evidence that IGF-1 ablation in DRG neuron cultures attenuated background neurite outgrowth and that IGF-1 overexpression promoted neurite outgrowth and mitochondrial respiration. We also found a more than twofold enrichment of binding of the transcription factors NFAT1 and CEBPβ to the IGF-1 promoter in DRG tissue from control rats compared with STZ-diabetic rats. CEBPβ and NFAT1 could both increase the mRNA levels of IGF-1 to elevate bioenergetics and neurite outgrowth of cultured DRG neurons. Taken together, our data suggest that DRG neurons synthesize and secrete IGF-1 that acts as an autocrine/paracrine neurotrophic factor playing a key role in neuronal maintenance and axonal outgrowth. These effects of IGF-1 developed over a 1–2 day period and compared with in vivo studies where IGF-1 or insulin was injected locally once per day to muscle or skin over a 2–4 day period. This resulted in elevated local sprouting of motor and sensory axons, respectively [37, 38]. This autocrine/paracrine support system is suppressed by hyperglycemia and may contribute to the pathogenesis of diabetic neuropathy.

Within the DRG, we found particularly high levels of IGF-1 in the TH and NP + ve sub-populations of small sensory neurons. The tyrosine hydroxylase (TH) cluster includes type-C low-threshold mechanoreceptors (C-LTMRs) that express high levels of the GDNF receptor and are implicated in the pleasurable component of touch and vibration [39, 40]. The non-peptidergic (NP) clusters are also C fibers that mostly express isolectin IB4 and are responsive to inflammatory itch and pruritus. The accumulation of endogenous IGF-1 protein against both sides of a pair of constricting ligatures of the sciatic nerve confirms a prior report [41] that IGF-1 undergoes both anterograde and retrograde fast axonal transport within the peripheral projections of primary afferents. Whether the IGF-1 undergoing retrograde transport represents endogenous protein that is being returned after anterograde transport to terminals or exogenous material that has been taken up at the primary afferent terminals remains unclear. The IGF-1 synthesized in these DRG neuronal sub-populations is presumably transported and released peripherally to provide trophic support for nerve endings and/or innervated target tissues such as epidermal keratinocytes, which express the IGF-1 receptor and in which the IGF-1/IGF-1 receptor axis regulates skin development and protects against damage by ultraviolet light [42, 43]. While we did not confirm axonal transport of IGF-1 along the central projections of primary afferents, the small unmyelinated TH and NP + ve population that expresses IGF-1 terminates in the superficial laminae of the spinal dorsal horn, a region associated with pain processing. Immunostaining for both the IGF-1 receptor and insulin receptor substrate 1 (IRS-1), the major downstream effector of IGF-1 receptor signaling, has been reported in laminae I–III [44, 45]. This raises the possibility that IGF-1 released into the superficial dorsal horn of the spinal cord may provide local trophic support and/or modulate pain processing, as has been suggested by studies using intrathecal delivery of exogenous IGF-1 [46].

Previous work has demonstrated that adult DRG neurons can function independent of exogenous neurotrophic growth factor support through expression of endogenous brain-derived neurotrophic factor (BDNF) [47]. Blockade of endogenous BDNF expression triggered 35% cell death in cultured adult DRG neurons. Upregulation of autocrine interleukin-6 (IL-6) was cAMP-dependent and promoted nerve regeneration after lesioning of the peripheral branch of DRG neurons [48]. In our present study, upregulation of endogenous IGF-1 production or its signaling augmented neurite outgrowth and mitochondrial respiration confirming the critical role of autocrine IGF-1 in maintaining the phenotype of adult sensory neurons in vitro. In addition, the low-dose growth factor cocktail (LDGF: NGF, GDNF and NT-3) that promoted neurite outgrowth and mitochondrial respiration was mediated via endogenous IGF-1 based upon the inhibitory effect of siRNA to IGF-1 (Supplemental Figure 2). Which neurotrophic factor in this cocktail is mediating this effect remains unclear, but it is worth noting that NGF treatment induces BDNF gene expression in a subpopulation of rat sensory neurons expressing the trkA NGF receptor sub-unit [49]. It is also feasible, given the proclivity of CEBPβ, for this transcription factor to regulate expression of multiple cytokines. Modulation of such proteins could also impact neurite outgrowth. For example, IL-6 expression, which is expressed endogenously by sensory neurons and can regulate axonal outgrowth in vivo and in vitro [50, 51], is under CEBPβ control. However, CEBPβ-dependent elevation of neurite outgrowth was completely inhibited by siRNA to IGF-1 (Fig. 7D), strongly supporting the notion that CEBPβ signaled uniquely via up-regulation of IGF-1 expression under these defined cell culture conditions.

We also characterized two transcription factors, CEBPβ and NFAT1, which elevated neurite outgrowth and bioenergetics through upregulation of endogenous IGF-1. CEBPβ is a transcription factor that can bind to DNA as a homodimer or heterodimer in combination with related proteins CEBPα, CEBPγ and CEBPδ [52]. CEBPβ is phosphorylated and activated by Ca^2+^/calmodulin-dependent protein kinase II (CaMKII) [53] and mitogen-activated protein kinase (MAPK) kinase kinase 1 (MKK1) [54]. This transcription factor can be inhibited by several upstream enzymes, such as protein kinase C (PKC) [55], protein kinase A (PKA) [55] and glycogen synthase kinase-3β (GSK-3β) [56]. The NFAT family of transcription factors was first identified as regulator of the gene expression of cytokines [57] and played a role in the immune response [58, 59]. Upstream enzymes, such as the serine/threonine phosphatase calcineurin, dephosphorylate NFAT transcription factors and induce translocation to the nucleus, whereas MAPK, PKA and GSK-3 re-phosphorylate and inactivate NFATs [60–62]. Reduced enrichment of these transcription factors at the *Igf-1* promoter (Fig. 5B) in DRG from STZ-diabetic rats may be due to transient changes in regulation. The most sensitive promoter region for binding is the region that is closest to the core promoter where RNA polymerase II binds and other regulatory elements, such as enhancers and co-activators, play a role [63, 64]. This might explain why elevated DNA methylation and reduced CEBPβ binding were observed in these regions (promoter region 5) of *Igf1* gene under the diabetic conditions in our study. However, we cannot rule out the potential involvement of other transcriptional or epigenetic inhibitory mechanisms.

Our data suggest that long-term epigenetic modification of the *Igf-1* gene was not responsible for the binding of these transcription factors since (1) the diabetes-induced downregulation of IGF-1 was readily reversible with a return to normoglycemia, blockade of aldose reductase activity by sorbinil, or treatment with IGF or LDGF (Fig. 2G–I, Supplemental Figure 2), and (2) our DNA methylation data for the IGF-1 promoter in DRG revealed no significant alterations under diabetic conditions, although there was an increase at cpg5 (Supplemental Figure 5C). However, it remains plausible that our DNA methylation analysis of the whole DRG may have missed differential effects of diabetes on specific sub-populations of neurons in the DRG. Laser capture microdissection could be used in future studies to compare DNA methylation status of specific sub-populations of sensory neurons and the effect of diabetes. Hyperglycemia and induction of polyol pathway activity trigger dysregulation of a range of critical kinases and phosphatases in a range of cell types. Inhibition of aldose reductase, the rate limiting enzyme of the polyol pathway, via sorbinil treatment reversed the inhibitory effect of the diabetic state on IGF-1 mRNA expression. Diabetes-induced activation of the polyol pathway and PKC leads to MAPK pathway activation and phosphorylation of several important transcription factors that control intracellular signaling and gene expression [65, 66]. Hyperglycemia-induced activation of PKC and/or suppression of CaMKII and calcineurin might therefore contribute to impaired activity and binding of NFAT1 and CEBPβ to target the *Igf1* gene promoter. In addition, polyol pathway activity can cause oxidative stress at multiple sites in neurons leading to production of methylglyoxal [67] and a range of advanced glycation end-products [68] that could potentially modify protein interactions at the CEBPβ promoter and triggering suppression of expression. Reduced IGF-1 expression in DRG was also observed in the pre-diabetic HFD mouse model, suggesting hyperglycemia per se was not a sole trigger of reduced CEBPβ function. However, in rat and mouse models of prediabetes, oxidative stress also develops in nerve tissue and contributes to neuropathy [69, 70]. This suggests oxidative stress may be a primary cause of suppression at the IGF-1 promoter.

The role of CEBPβ in the nervous system includes enhancing expression of neurokinin A and substance P, and modulating the biosynthesis of acetylcholine in neuronal cell lines and striatal neurons [71, 72]. NFATc2 (NFAT1) and NFATc4 also play a role in adipogenesis and glucose/insulin homeostasis, so that they serve as factors regulating metabolic processes and adipokine gene transcription [73]. Neurotrophins, such as NGF and BDNF, require NFATc4 activation to promote axonal outgrowth in embryonic DRG neurons derived from mice. Mice lacking NFATc2, c3 and c4 are deficient in axonal outgrowth despite no or little defect in neuronal survival and differentiation [74]. NFAT and CEBPβ cooperation is required for binding to composite elements on IGF-2 and PPARγ2 regulatory regions [75]. No effect of NFAT1 or CEBPβ on mitochondrial function in the nervous system has previously been reported. Here, we show that NFAT1 or CEBPβ overexpression increased glycolysis and mitochondrial respiration in sensory neurons, and that this was IGF-1-dependent. NGF regulation of mitochondrial function and neurite outgrowth in sensory neurons might therefore be mediated through induction of NFAT and CEBPβ transcription factors and activation of endogenous IGF-1 expression [76].

Other than neurons, a high level of IGF-1 mRNA was also present in SGCs. Multiple SGCs surround one neuron and form a unit that responds synchronously to environmental stimuli and pathological conditions, such as neuropathic pain and nerve injury [77]. For example, axotomy of sciatic nerve in rodents elevated fibroblast growth factor-2 (FGF-2) expression in SGCs in the DRG [78]. Moreover, peripheral nerve injury in mice induced fatty acid synthesis and peroxisome proliferator-activated receptor (PPARα) signaling in SGCs, while knockout of fatty acid synthase or PPARα inhibition specifically in SGCs impaired subsequent axonal regeneration following nerve crush [79]. Finally, GDNF mRNA increased in SGCs of L4/5 DRGs after chronic sciatic nerve injury in rats and remained at high levels for 5 months [80]. It is possible that IGF-1 secretion from SGCs in response to stress can accelerate the repair and regeneration process within the DRG by providing neurotrophic support in a paracrine fashion to local neurons. As well as providing a response to nerve injury, there may also be ongoing IGF-1-mediated support of sensory neurons by SGC that is disrupted by insults such as diabetes and thus contributes to the pathogenesis of diabetic neuropathy. Activation of SGC, as illustrated by increased expression of GFAP and the purinergic P2Y12 receptor in diabetic rodents [81] suggests that SGCs are under metabolic stress. This could be secondary to a hyperglycemia-induced increase in polyol pathway activity, as SGCs are the major site of aldose reductase expression in DRG [82], thereby providing a mechanism by which aldose reductase inhibition was effective in restoring IGF-1 expression and release in DRG of diabetic rodents and cells exposed to hyperglycemia.

We previously demonstrated the beneficial consequence of insulin and IGF-1 treatment in preventing diabetic neuropathy in STZ-induced diabetic rats [19, 83, 84]. The present study extends these findings to highlight the role of endogenous IGF-1 in regulation of bioenergetics and neurite outgrowth in DRG neurons. Transcription factors CEBPβ and NFAT1 acted by regulating IGF-1 transcription to augment mitochondrial respiration and axonal regeneration in cultured DRG neurons. Suppression of endogenous IGF-1 in DRG cultures impedes background axonal outgrowth, and depletion of IGF-1 by a hyperglycemia-driven increase in polyol pathway activity which may contribute to neurodegeneration and suppress neurite outgrowth. Despite accumulating evidence supporting the value of local or systemic IGF-1 administration for treatment of diabetic neuropathy and other neurodegenerative disorders, such as Alzheimer’s, ALS and Rett syndrome, relatively little is still known about the role of endogenous IGF-1 in peripheral tissues. The Diabetes Control and Complications trial (DCCT) demonstrated that intensive insulin therapy in persons with type 1 diabetes leads to a lower incidence of distal symmetrical polyneuropathy. However, despite careful control of hyperglycemia, 25% of patients on intensive insulin treatment still presented with neuropathy at study end [85]. In the context of type 2 diabetes, metformin therapy remains first-line treatment and clearly prevents development of complications. However, the impact on peripheral neuropathy remains complex with some incidence of increased neuropathy due to suppression of vitamin B12 levels [86]. Thus, identifying novel therapeutic approaches remains a strong clinical imperative. Our data support the idea that IGF-1–LNP may provide a path to safe and effective gene therapy in neurodegenerative disorders, while the CEBPβ and NFAT1 transcription factors could also represent promising targets manipulating endogenous IGF-1 production for the treatment of diabetic neuropathy.

### Supplementary Information

Below is the link to the electronic supplementary material.

Supplementary file1 (DOCX 41 KB)

Supplementary file2 Supplemental Fig. 1: Endogenous IGF-1 was detectable in the liver, reduced in diabetic rodents, and expressed at a higher level in brain cortex and DRG tissue vs. sciatic nerve tissue. In (A), liver sections from control rats underwent RNA FISH assay for IGF-1 mRNA detection and localization. The punctate pattern of IGF-1 staining represents its mRNA. Images are magnified for clarification. As a control, tissue sections and cells were exposed to RNase enzyme before hybridizing with IGF-1 probes. In (B-D), DRG, sciatic nerve and brain cortex tissues were obtained from control rats and underwent (B) Northern blotting or (D) Real-Time PCR assay. In (C), 2 µg RNA was run on 1.2% TAE-agarose gel for quality control in (B) and normalization purposes in (D). IGF-1 fluorescent probes were used for hybridization in (B). In (E), PCR products from (D) were run on 1.2% TAE-agarose gel to compare the band intensities. In (F), liver tissues from control (Ctrl), hIGF-1-treated (STZ-Db + hIGF-1) and untreated STZ-diabetic (STZ-Db) rats were homogenized and underwent ELISA for IGF-1 detection. In (G), Volcano plot of upregulated and downregulated genes in the transcriptome of DRG of mice on Western diet vs. normal chow is illustrated (n = 2 biological replicates, biological replicates are 9 DRG from 3 mice). In red significant hits, q value < 0.05, green dots in circle represent igf1 and igf1r as indicated. Data (A-F) are mean ± SEM of N = 3–4; * = p < 0.05 or ** = p < 0.01 or *** = p < 0.001; analyzed by Student’s *t* test or one-way ANOVA with Tukey’s post hoc test. (TIF 29807 KB)

Supplementary file3 Supplemental Fig. 2: IGF-1 neutralizing antibody modulated the neurotrophic factor-dependent elevation of mitochondrial respiration and neurite outgrowth. DRG tissues from STZ-diabetic (Db) rats were cultured in the presence of low-dose growth factors (LDGFs: NGF, GDNF and NT-3) and/or IGF-1 neutralizing antibody. In (A), OCR data were normalized to total protein in mg in each treatment group. In (B), sensory neurons larger than 50 microns in diameter (traditionally classified as large diameter DRG neurons) were selected for analysis since they showed stronger phenotype. Data are mean ± SEM of N = 3–5; * = p < 0.05 or ** = p < 0.01 or *** = P < 0.001 or **** = P < 0.0001; analyzed by one-way ANOVA with Dunnett’s or Tukey’s post hoc test. (TIF 14747 KB)

Supplementary file4 Supplemental Fig. 3: IGF-1-overexpressing plasmid enhanced glycolysis and mitochondrial respiration in human HEK293 cell line. HEK293 cells were serum-starved for 6 h, transfected with 2ug hIGF-1 (transcript variant 4)-overexpressing plasmid (pIGF-1) or control GFP plasmid (ctrl). In (A and B), mitochondrial OCR was measured in live cells after 36 h. In (C-F), glycolysis parameters were calculated. Total protein in mg was used to normalize OCR and ECAR data. Data are mean ± SEM of N = 4–5; ** = p < 0.01; analyzed by Student’s *t* test. (TIF 35562 KB)

Supplementary file5 Supplemental Fig. 4: Bioinformatic screening of IGF-1 promoter for transcription factor binding and ChIP assay validation. Four transcript variants of rat IGF-1 gene were screened for transcription factor binding on the promoter. The two top inclusive transcription factors, (A) NFAT1 and CEBP-β, were chosen for further experiments. In (B), chromatin fragmentation optimization was performed prior to ChIP experiments. In (C), the validity of PCR products from five promoter regions were tested on an agarose gel. Data are mean ± SEM of N = 5; * = p < 0.05; analyzed by Student’s *t* test. (TIF 24806 KB)

Supplementary file 6 Supplemental Fig. 5: The level of NFAT1 and CEBPβ proteins, and IGF-1 promoter DNA methylation was not significantly changed under diabetic conditions. DRG tissues from control (Ctrl), hIGF-1-treated (Db + hIGF-1) and untreated diabetic (Db) rats were homogenized and underwent (A-B) Western blotting for NFAT1 and CEBP-β (LAP1 and LAP2 isoforms) proteins. Total ERK band intensity was used for normalization. In (C), DNA methylation flanking transcription factor (NFAT1 and CEBPβ) binding sites on IGF-1 promoter was quantified in percent in DRG tissues from control and diabetic rats. Data are mean ± SEM of N = 3–8 animals; analyzed by Student’s *t* test or one-way ANOVA with Tukey’s post hoc test. (TIF 30799 KB)

Supplementary file7 Supplemental Fig. 6: Validation of GFP, IGF-1, CEBPβ and NFAT1-overexpressing plasmids in HEK293 cells or DRG neurons. HEK293 cells were cultured and transfected with GFP, IGF-1, CEBPβ or NFAT1 followed by Western blotting or qRT-PCR. In (A), GFP-HisTag was detected using blotting against GFP antibody. In (B and C), two coding regions of IGF1-HisTag plasmid were amplified using qRT-PCR and were run on an agarose gel. CEBPβ and NFAT1 overexpression was confirmed both in (D) DRG culture and (E and F) HEK293 cell line culture using Western blotting. Data are mean ± SEM of N = 4; analyzed by Student’s *t* test. (TIF 39242 KB)

Supplementary file8 Supplemental Fig. 7: CEBPβ-overexpressing plasmid increased mitochondrial respiration but not glycolysis. In (A and B), DRG neurons from control (ctrl) rat were transfected with GFP (ctrl) or 0.5ug CEBPβ-overexpressing plasmids and underwent mitochondrial respiration assay. In (C-F), HEK293 cells were serum-starved for 1 day, transfected with 0.5ug CEBPβ-overexpressing plasmid or control GFP plasmid (ctrl). In (C and D), mitochondrial OCR was measured in live cells after 36 h. In (E and F), glycolysis parameters were calculated. Total protein in mg was used to normalize OCR and ECAR data. Data are mean ± SEM of N = 4–5; * = p < 0.05 or ** = p < 0.01; analyzed by Student’s *t* test. (TIF 36158 KB)

Supplementary file9 Supplemental Fig. 8: NFAT1-overexpressing plasmid upregulated mitochondrial respiration and glycolysis. In (A and B), DRG neurons from control (ctrl) rat were transfected with GFP (ctrl) or 0.5ug NFAT1-overexpressing plasmids and underwent glycolysis assay. In (C-F), HEK293 cells were serum-starved for 1 day, transfected with 0.5ug NFAT1-overexpressing plasmid or control GFP plasmid (ctrl). In (C and D), mitochondrial OCR was measured in live cells after 36 h. In (E and F), glycolysis parameters were calculated. Total protein in mg was used to normalize OCR and ECAR data. Data are mean ± SEM of N = 4–5; ** = p < 0.01 or *** = p < 0.001; analyzed by Student’s *t* test. (TIF 37811 KB)

Supplementary file10 Supplemental Fig. 9: NFAT1 overexpression increased ATP5a level and mitochondrial respiration in DRGs, and IGF-1 knock-down modulated the elevated OCR level. DRG neurons from control (ctrl) or STZ-diabetic (Db) rats were cultured, transfected with NFAT1 or GFP plasmids or siIGF1–LNP. In (A and B), DRG tissues from control rats were subjected to Western blotting for mitochondrial ETS proteins. In (C and D), DRGs from Db rats were cultured, transfected with either GFP plasmid or NFAT1 plasmid and/or siIGF–LNP for 36 h and subjected to mitochondrial respiration analysis. Total protein in mg was used to normalize OCR data. Data are mean ± SEM of N = 4–5; * = p < 0.05; analyzed by Student’s *t* test or one-way ANOVA with Tukey’s post hoc test. (TIF 26670 KB)

## Data Availability

All data analyzed during this study are included in this published article [and its supplementary information files]. The raw data and datasets generated during the current study are available from the corresponding author on reasonable request.
