# Supplementary material for: CEBPβ regulation of endogenous IGF-1 in adult sensory neurons can be mobilized to overcome diabetes-induced deficits in bioenergetics and axonal outgrowth
Source: Cell Mol Life Sci. 2022 Mar 17;79(4):193. doi: 10.1007/s00018-022-04201-9 (PMC8930798; doi:10.1007/s00018-022-04201-9)
Supplement: Supplementary file 1 — Supplementary file1 (DOCX 41 KB) [file 18_2022_4201_MOESM1_ESM.docx]

**Supplementary Methods for *Cellular and Molecular Life Sciences***

CEBPβ regulation of endogenous IGF-1 in adult sensory neurons can be mobilized to overcome diabetes-induced deficits in bioenergetics and axonal outgrowth

**Mohamad-Reza Aghanoori^1,2,*^, Prasoon Agarwal^2,3^, Evan Gauvin^1^, Raghu S. Nagalingam^4,5^, Raiza Bonomo ^6^, Vinith Yathindranath^7^, Darrell R. Smith^1^, Yan Hai^8^, Samantha Lee^8^, Corinne G. Jolivalt^9^, Nigel A. Calcutt^9^, Meaghan Jones^8^, Michael P. Czubryt^4,5^, Donald W. Miller^7^, Vernon W. Dolinsky^2,3^, Virginie M. Aubert^6^ and Paul Fernyhough^1,2^**

*^1^Division of Neurodegenerative Disorders, St. Boniface Hospital Albrechtsen Research Centre, Winnipeg, MB, Canada*

*^2^Dept of Pharmacology & Therapeutics, University of Manitoba, MB, Canada*

*^3^Children's Hospital Research Institute of Manitoba, University of Manitoba, MB, Canada*

*^4^Department of Physiology and Pathophysiology, Rady Faculty of Health Sciences, University of Manitoba, MB, Canada*

*^5^Institute of Cardiovascular Sciences, St. Boniface Hospital Albrechtsen Research Centre, MB, Canada*

*^6^Cellular and Molecular Department, Stritch School of Medicine, Loyola University Chicago, Chicago, USA*

*^7^Kleysen Institute for Advanced Medicine, University of Manitoba, MB, Canada*

*^8^Department of Biochemistry and Medical Genetics, Rady Faculty of Health Sciences, University of Manitoba, MB, Canada*

*^9^Department of Pathology, UCSD, La Jolla, CA, USA*

**Corresponding author:* Mohamad-Reza Aghanoori, Dept of Medical Genetics, University of Calgary 3330 Hospital Drive NW Calgary, Alberta, T2N 4N2, Cell: (204) 894-5869; E-mail: [mohammadreza.aghanoo@ucalgary.ca](mailto:mohammadreza.aghanoo@ucalgary.ca)

**Materials and methods:**

**Animals**

Male Sprague-Dawley rats (275–325 g) were used as a model of type 1 diabetes after delivery of a single intraperitoneal injection of 90 mg/kg Streptozotocin (STZ) (Sigma, St Louis, MO, USA) and were compared with age-matched control rats. Adult Zucker diabetic fatty (ZDF) rats and *db/db* mice were used as models of type 2 diabetes in this study. Diabetic rats developed neuropathy after 3 months of STZ injection. A subgroup of STZ-diabetic animals received 20µg hIGF-1 peptide (peritoneal injection) thrice weekly for 11 weeks after 3 months of diabetes. Fasting blood glucose concentration was monitored half-way through the injection period and at study end using an AlphaTRAK glucometer (Abbott Laboratories, Illinois, USA) to ensure that our treatment did not affect hyperglycemia. At the study end, blood glucose, glycated hemoglobin (HbA1c Multi-test system, HealthCheck Systems, Brooklyn, NY, USA) and body weight were recorded before tissue collection. The neuropathy status of these animals has been described previously [31]. For western diet studies, control C57BL/6J was fed normal Chow (NC) (Teklad LM-485), while the experimental groups were fed Western Diet (WD) (TD88137, Teklad Diets; 42%kcal from fat, 34% sucrose by weight, and 0.2% cholesterol total - Envigo, Indiana, USA) for 14 weeks when exhibited glucose intolerance and neuropathy. Animal procedures were approved by the University of Manitoba Animal Care Committee and followed Canadian Council of Animal Care (CCAC) rules, National Institutes of Health and the Loyola University Chicago Institutional Animal Care and Use Committee.

**Adult DRG sensory neuron and HEK293 cell culture**

DRGs were isolated from adult male Sprague-Dawley (300-350g) rats and dissociated using previously described methods [1]. Neurons were cultured in no-glucose Hams F12 media supplemented with Bottenstein’s N2 without insulin (0.1 mg/ml transferrin, 20 nM progesterone, 100 µM putrescine, 30 nM sodium selenite 0.1 mg/ml BSA; all additives were from Sigma, St Louis, MO, USA; culture medium was from Caisson labs, USA). DRG neurons from control rats were cultured in the presence of 5 mM D-glucose and DRG neurons derived from STZ-induced diabetic rats with 25 mM D-glucose. No neurotrophins or insulin were added to any DRG cultures. The HEK293 (ATCC CRL-1573, Virginia, USA) cell line was cultured in DMEM/F12 (1:1) media containing 10% FBS. The following pharmacological inhibitor was used in this study: Sorbinil, an inhibitor of aldose reductase (Sigma, St Louis, MO, USA)

**Preparation of LNP-siRNA and LNP-pDNA for IGF-1 knockdown or overexpression**

siRNA and pDNA encapsulated LNPs were prepared following the microfluidics mixing method. The lipid mix supplied with the Neuro9 kit was used for the formulation. A mixture of siRNA-29, 5’-GCUGAAGCCUACAAAGUCAtt-3’ (siRNA ID: s127929, Thermo Scientific, Pittsburgh, PA, USA) and siRNA-31, 5’-GAAGUACACUUGAAGAACAtt-3’ (siRNA ID: s127931, Thermo Scientific, Pittsburgh, PA, USA) specific to rat IGF-1, and a scrambled siRNA (Cat #:4635, Thermo Scientific, Pittsburgh, PA, USA) were used in the formulation. GFP (AG13105-CH, Sino Biological Inc., Beijing, China), IGF-1 (HG29626-NH, Sino Biological Inc., Beijing, China) and CEBPβ (a gift from Peter Johnson, Addgene plasmid # 12557; http://n2t.net/addgene:12557; Massachusetts, USA) overexpressing plasmids were used for the LNP-pDNA formulations. For the microfluidic mixing, 1 volume of the supplied lipid mix (organic phase) and 3 volumes of the siRNA in the supplied formulation buffer (aqueous phase) were micromixed using a NanoAssemblr Benchtop instrument (Precision NanoSystem, Vancouver, BC). In all cases, the siRNA and pDNA concentration of 0.5 mg (in aqueous phase) was used for 1 mL of the lipid mix (organic phase). A flow rate ratio (FRR) of 1:3 (organic:aqueous) and a total flow rate (TFR) of 12 mL/min was used to yield LNP-siRNA and LNP-pDNA. The obtained LNP formulations were diluted (50-times) in supplied wash buffer and purified/concentrated using a centrifugal filter (2000×g, MWCO 10000) to its original volume. The concentration of the encapsulated siRNA and pDNA in the LNP formulations was measured using Thermo Scientific NanoDrop spectrophotometer (Madison, WI). For transfection (36h), 200-600ng LNP-pDNA and/or 100ng LNP-siRNA were used for each well of a 24-well plate in the corresponding experiments.

**Quantitative Western blotting**

Rat DRG neurons were harvested from culture or isolated intact from adult rats or mice and then homogenized in ice-cold RIPA buffer containing: 25mM Tris pH=8, 150 mM NaCl, 0.1% SDS, 0.5% sodium deoxycholate, 1% Triton X-100 and protease phosphatase inhibitors. Proteins (2-20 μg total protein/lane) were resolved and separated via 4-20% sodium dodecyl sulphate-polyacrylamide gel electrophoresis (SDS-PAGE). The proteins were subsequently transferred to a nitrocellulose membrane (Bio-Rad, CA, USA) using Trans-Blot Turbo Transfer System (Bio-Rad, CA, USA) and immunoblotted with specific antibodies against pAkt S473 (1:1000, Santa Cruz Biotechnology, Texas, USA), total Akt (1:1000, Abcam, Cambridge, MA, USA), IGF-1Rβ (1:1000, Santa Cruz Biotechnology, Texas, USA), NFAT1 (1:1000, Abcam ab2722, Cambridge, MA, USA), CEBPβ (1:1000, Abcam ab32358, Cambridge, MA, USA) total OXPHOS (1:2000, MitoSciences, Abcam, Cambridge, MA, USA) and total ERK (1:1000, Santa Cruz Biotechnology, Texas, USA). Total protein bands were captured by chemiluminescent imaging of the blot after gel activation (TGX Stain-Free™ FastCast Acrylamide Solutions, Bio-Rad, CA, USA) in addition to the use of T-ERK levels for target protein normalization (to adjust for loading). The secondary antibodies were HRP-conjugated goat anti-rabbit IgG (H+L) or goat anti-mouse IgG (H+L) from Jackson ImmunoResearch Laboratories, PA, USA. The blots were incubated in ECL Advance (GE Healthcare) and imaged using a Bio-Rad ChemiDoc image analyzer (Bio-Rad, CA, USA).

**ELISA assay**

Homogenized tissues or collected media from DRG cultures were assayed using the Mouse/Rat IGF-1 Quantikine ELISA kit (R&D Systems, Minnesota, USA) according to the kit instructions.

**RNA isolation, cDNA library construction and Illumina sequencing**

Total RNA was extracted from DRG of normal chow (NC) or western diet (WD) fed mice with the Arcturus PicoPure RNA isolation kit (Applied Biosystems). Two biological replicates (6 lumbar DRG per replicate from 3 mice) were used for each group. Total RNA was quantified by Qubit and assessed for quality on an Agilent Bioanalyzer using Total RNA Pico Chip. Total RNA samples that passed QC were used as input for library construction. Full-length cDNA synthesis and amplification were carried out with the Clontech SMART-Seq v4 Ultra Low Input RNA Kit. Subsequently, Illumina sequencing libraries were prepared from the amplified full-length cDNA with the Nextera XT DNA Library Preparation Kit. Prior to sequencing, the prepared libraries were quantified with Qubit and validated on a Bioanalyzer with a High Sensitivity DNA chip. The sequencing of the libraries was conducted on an Illumina NextSeq 500 NGS System. Single 75 bp reads were generated with dual indexing. RNA-seq Analysis was done with STAR and DESeq2. The quality of reads, in FASTQ format, was evaluated using FastQC. Reads were trimmed to remove Illumina adapters from the 3’ ends using cutadapt. Trimmed reads were aligned to the Mus musculus genome (mm10) using STAR. Read counts for each gene were calculated using htseq-count in conjunction with a gene annotation file for mm10 obtained from Ensembl (http://useast.ensembl.org/index.html). Normalization and differential expression were calculated using DESeq2 that employs the Wald test. For determining significance, a q value or FDR (false discovery rate)- adjusted p value < 0.05 was used to obtain all genes regulated. No cut–off was applied to keep all significantly regulated genes in the analysis.

**Real-Time PCR**

RNA was extracted from cultured neurons or previously frozen tissue samples using TRIzol® Reagent (Invitrogen, California, USA). Complementary DNA (cDNA) was synthesized from RNA samples by using the iScript™ gDNA Clear cDNA Synthesis Kit (Bio-Rad, CA, USA) according to the manufacturer's instructions. Quantitative real-time PCR (QRT-PCR) was performed using iQ™ SYBR® Green Supermix (Bio-Rad, CA, USA) or Bright Green Master mix (Abmgood Co., Richmond, Canada) compatible with the iQ5 Cycler machine (Bio-Rad, CA, USA). The ΔΔCt method was used to quantify gene expression. The mRNA level of GAPDH, 18s rRNA and B2m were used for normalization.

**Chromatin immunoprecipitation (ChIP) assay**

Transcription factor binding on rat IGF-1 promoter was assessed to select the highest hits prior to ChIP assay. Briefly, all four transcript variants of IGF-1 gene from Rat genome version 6 were extracted using BioMart-Ensembl. The DNA sequence of 1200 bases upstream of all transcription initiation sites was retrieved for transcription factor binding assessment. Then, Gene Promoter Miner (<http://gpminer.mbc.nctu.edu.tw/index.php>) was used to predict transcription factors that bind to rat IGF-1 promoter from which 2 highest hit transcription factors NFAT1 and CEBPβ were selected for ChIP analysis. For this purpose, DRG tissues from rats were dissected, weighed and underwent ChIP analysis assay using the ChromaFlash™ High-Sensitivity ChIP Kit (Catalog # P-2027, Epigentek, Farmingdale, NY, USA). Briefly, 100 mg amounts of DRG tissues were cut into small pieces, cross-linked using 1% formaldehyde for 15 min, quenched by 1.25mM glycine, centrifuged, washed and homogenized in lysis buffer using a Dounce homogenizer. The chromatin pellet was resuspended in ChIP buffer and sonicated three times (15sec each) with 40sec intervals. Chromatin from this step was added to pre-incubated antibodies (non-immune IgG or CEBPβ antibody-ab32358 or NFAT1 antibody-ab2722 by Abcam, Cambridge, MA, USA) in each assay strip well and incubated on an orbital shaker for 2 hours. A portion of this chromatin (10% dilution) was also saved to use as input for Real-Time PCR experiments as control. Following stringent washes, DNA fragments were collected in RNase A-Proteinase K-containing DNA releasing buffer. Different regions of rat IGF-1 promoter using specific primers and DNA samples from each group were used for Real-Time PCR. Finally, fold enrichment was calculated using the formula FE=2^(IgG CT - sample CT)^ to test if there were binding sites for NFAT1 or CEBPβ on the IGF-1 promoter.

**RNA FISH**

We designed 28 oligonucleotide probes, 18-26 bp in length, spanning the whole rat IGF-1 mRNA. They were fluorescently labeled with Quasar 570 (Stellaris RNA FISH, Biosearch Technologies, Petaluma, CA, USA) so that we could image using a Carl Zeiss Axioscope-2 upright fluorescence microscope equipped with AxioVision3 software. To visualize IGF-1 mRNA inside the cultured DRGs, we used the protocol for adherent cells (Stellaris RNA FISH, Biosearch Technologies, Petaluma, CA, USA). Briefly, cells seeded on coverslips were fixed with 3.7% formaldehyde for 10 min, permeabilized with 70% ethanol for 1 hour at 4$^{\circ}$C and washed. Then, coverslips were mounted onto a 100µl hybridization buffer (made of sodium citrate buffer, formamide, salmon sperm DNA and dextran sulfate in nuclease-free water) containing 125nM probe mixture in a humidified chamber for 16 hours at 37$^{\circ}$C. Coverslips were washed in wash buffer A (made of sodium citrate buffer and formamide in nuclease-free water) for 30min at 37$^{\circ}$C, stained with Hoechst and washed in buffer B (made of sodium citrate buffer, Tween-20 and formamide in nuclease-free water) for 5min prior to mounting on slides for imaging. To visualize IGF-1 mRNA in DRG and liver tissues, we snap froze the OCT-embedded tissues on dry ice, sectioned at a thickness of 10µm using a cryostat and followed the protocol for frozen tissues (Stellaris RNA FISH, Biosearch Technologies, Petaluma, CA, USA). Finally, we captured 20 images per group with a magnification of 63X. As a negative control we used a culture/section group without any probe or treated with RNase A (50 µg/mL) for 30 min at 37 °C, prior to the hybridization step.

**Northern blotting**

RNA was extracted from DRG, sciatic nerve and brain tissues from rat using TRIzol® Reagent (Invitrogen, California, USA). A modified protocol from the Hackett lab (https://cbs.umn.edu/hackett-lab/protocols/northern-blotting) for Northern blotting and hybridization was used. In brief, twenty micrograms (20µg) of extracted RNAs were mixed in RNA sample buffer containing 62.5% formamide, 1.14M formaldehyde, 1.25X TAE buffer, 200 µg/ml Xylene Cyanol FF and 200 µg/ml bromophenol blue, heated at 65°C for 15min and kept on ice to prevent renaturation of RNA. Denaturing gels were made by adding 1.2g agarose into 72ml nuclease-free water, heated and mixed with 10ml 10X TAE buffer and 18ml 37% formaldehyde (12.3M). Samples were run on the gel at 5V/cm in 1X TAE buffer. Gel was washed in RNase-free water and in transfer buffer (20X sodium citrate buffer) for 20min. In a pool of 20X transfer buffer, RNAs were transferred from the gel to nitrocellulose overnight, baked in oven at 80°C for 2 hours and subjected to hybridization (IGF-1 fluorescent probe mix-see RNA FISH above) for 16 hours at 42°C. The nitrocellulose filter was washed in 0.2X sodium citrate buffer containing 0.1% SDS for multiple times before visualization using a Bio-Rad ChemiDoc image analyzer (Bio-Rad, CA, USA). Two micrograms (2µg) of total RNA was used and run on a 1.2% agarose gel to visualize 5S, 18S and 28S rRNA from each tissue sample for normalization of Real-Time PCR and Northern blotting results.

**Site-directed mutagenesis on IGF-1 promoter and luciferase-reporter assay**

Rat IGF-1 gene promoter (about 1kb upstream to exon 1) was amplified using Q5 high fidelity DNA polymerase (Cat#: M0491G, New England Biolabs, Massachusetts, USA) with primers having HindIII and XhoI recognition sites at their 5’ ends. PCR product was treated with these restriction enzymes (New England Biolabs, Massachusetts, USA), ligated into pGL4.10 [luc2] (Promega, Wisconsin, USA) and transformed into DH5a cells according to the manufacturer’s instructions (New England Biolabs, Massachusetts, USA). Q5® Site-Directed Mutagenesis Kit (New England Biolabs, Massachusetts, USA) and 4 pairs of primers were used to make mutated binding sites for NFAT1 or CEBPβ transcription factors in the IGF-1 promoter part of the construct. Plasmids were purified from single colonies, sequenced for validation, and co-transfected with pcDNA 3.1(+) NFAT1 or pcDNA 3.1(-) mouse CEBPβ (LAP) (a gift from Peter Johnson, Addgene plasmid # 12557; http://n2t.net/addgene:12557 ; RRID:Addgene_12557) and a Renilla plasmid into HEK293 cell line. IGF-1 promoter-driven luciferase activity was measured and recorded as bioluminescent unit using Glomax-multi detection system (Promega, Wisconsin, USA). Emitted bioluminescent was normalized to Renilla emissions and plotted.

**Mitochondrial respiration and glycolysis assay in cultured neurons and HEK293 cells**

Mitochondrial oxygen consumption rate (OCR) was measured in live sensory neurons and HEK293 cells using the XF24 analyzer (Seahorse Biosciences, Billerica, MA, USA). In brief, DRG culture medium was changed 1hr before the assay to unbuffered DMEM (Dulbecco's modified Eagle's medium, pH 7.4) supplemented with 1mM sodium pyruvate, and 5 mM D-glucose in case of control or 25 mM D-glucose in case of diabetic rat used. Four mitochondrial complex inhibitors including oligomycin (1 µM), FCCP (1 µM) and rotenone (1 µM) + antimycin A (1 µM) were injected sequentially through ports in the Seahorse Flux Pak cartridges. Oligomycin acts as an irreversible ATP synthase inhibitor, FCCP as an uncoupler, rotenone as Complex I inhibitor, and antimycin A as an inhibitor of Complex III of the mitochondrial electron transport system. For glycolysis test, glucose (10mM), oligomycin (1µM) and 2-deoxy-glucose (2DG: a glucose analog) (50mM) were sequentially injected to achieve extracellular acidification rate (ECAR). After OCR and ECAR measurements, parameters such as basal respiration, maximal respiration, spare respiratory capacity, respiratory control ratio, glycolysis and glycolysis reserve were computed after data normalization to mg protein (DC protein assay, BioRad, USA). Therefore, OCR measures are presented as pmoles/min/mg protein and ECAR measures are presented as mpH/min/mg protein.

**Immunocytochemistry**

DRG neurons were cultured on glass coverslips and were fixed with 4% paraformaldehyde in PBS (pH 7.4) for 15 min at room temperature and permeabilized with 0.3% Triton X-100 in PBS for 5 min. Neurons were incubated with 5% BSA in PBS for 1 h and with neuron-specific β-tubulin III antibody (1:1000; from Sigma, St Louis, MO, USA) or IGF-1Rβ antibody (Santa Cruz Biotechnology, Texas, USA) overnight. Cells were incubated with Cy3-conjugated secondary antibody (1:1000, Jackson ImmunoResearch Laboratories Inc., PA, USA) for 1 h at room temperature following three washes with PBS. Coverslips were mounted on slides using VECTASHIELD antifade mounting medium with DAPI (Vectorlabs, inc. CA, USA) and imaged using a Carl Zeiss Axioscope-2 upright fluorescence microscope equipped with AxioVision3 software. Alternatively, LSM 510 confocal microscope (Carl Zeiss AG, Oberkochen, Germany) was used to image live cells transfected with tubulin-GFP plasmid in the Amaxa® Rat Neuron Nucleofector Kit (Lonza Inc., [Basel, Switzerland](https://www.google.ca/url?sa=t&rct=j&q=&esrc=s&source=web&cd=2&cad=rja&uact=8&ved=0ahUKEwjDmZ7u5fvZAhVDEawKHdcyD8sQjBAINTAB&url=https%3A%2F%2Fwww.lonza.com%2Fabout-lonza%2Fcompany-profile%2Flocations-worldwide%2Fbasel-switzerland.aspx&usg=AOvVaw2uQSCozP2jqsoYCaHoyM9D)) using Amaxa Nucleofector machine (Lonza Inc., Basel, Switzerland). To quantify neurite outgrowth, the fluorescent signal was collected as total pixel area for neurites and was measured by the high throughput NeurphologyJ plugin in ImageJ software after image enhancement. Total pixel area was normalized to number of cell bodies to calculate total neurite outgrowth per neuron.

**Pyrosequencing**

DNA was extracted from DRG tissues from diabetic and control rats and underwent bisulfite conversion per manufacturer’s instructions (Cat#D5020, Zymo Research, USA). Biotinylated primers and the pyrosequencing assays were designed using PyroMark Assay Design 2.0 (Qiagen, Inc.) software to cover 7 CpG sites on IGF-1 promoter. PCR and pyrosequencing performed as previously described [2]. Streptavidin-coated sepharose beads were bound to the biotinylated strand of the PCR product and were then washed and denatured to yield single-stranded DNA. Sequencing primers were then added for pyrosequencing per manufacturer’s instructions (Pyromark™ Q96 MD Pyrosequencer, Qiagen, Inc.).

**Single-cell and whole DRG RNA sequencing data acquisition and analysis**

Normalized expression level (RPM) data from single-cell RNA-Seq (scRNA-Seq) study of mouse DRG tissues was obtained from publicly available datasets (GSE59739 in GEO database) which were previously deposited by Usoskin et al. [3]. Then, the expression level of markers of each DRG subpopulation together with Igf1 and igf1r expression levels were extracted and analyzed. The markers for each DRG cell population used were as follows: NF cluster (myelinated neurons): neurofilament heavy chain (NEFH), parvalbumin (PVALB) and βIII-tubulin (TUBB3), PEP cluster (peptidergic nociceptors): substance P (TAC1), TrkA (NTRK1), calcitonin gene-related peptide (CALCA) and βIII-tubulin (TUBB3), NP cluster (nonpeptidergic nociceptors): purinergic receptor P2X 3 (P2RX3) and βIII-tubulin (TUBB3), TH cluster (Type C low-threshold mechanoreceptors): tyrosine hydroxylase (TH) and βIII-tubulin (TUBB3), SC cluster (Schwann cells): P75 receptor and myelin basic protein (MBP), SGC cluster (Satellite glial cells): glycogen synthase (GS) and S100 calcium binding protein B (S100B). Relative IGF-1 and IGF-1R expression levels were plotted to compare the mean and distribution of them in each DRG subgroup.

**References:**

1. Calcutt, N.A., et al., Selective antagonism of muscarinic receptors is neuroprotective in peripheral neuropathy. J Clin Invest, 2017. 127(2): p. 608-622.

2. Morin, A.M., et al., Maternal blood contamination of collected cord blood can be identified using DNA methylation at three CpGs. Clin Epigenetics, 2017. 9: p. 75.

3. Usoskin, D., et al., Unbiased classification of sensory neuron types by large-scale single-cell RNA sequencing. Nat Neurosci, 2015. 18(1): p. 145-53.
