# Supplementary figures and images for: CEBPβ regulation of endogenous IGF-1 in adult sensory neurons can be mobilized to overcome diabetes-induced deficits in bioenergetics and axonal outgrowth
Source: Cell Mol Life Sci. 2022 Mar 17;79(4):193. doi: 10.1007/s00018-022-04201-9 (PMC8930798; doi:10.1007/s00018-022-04201-9)

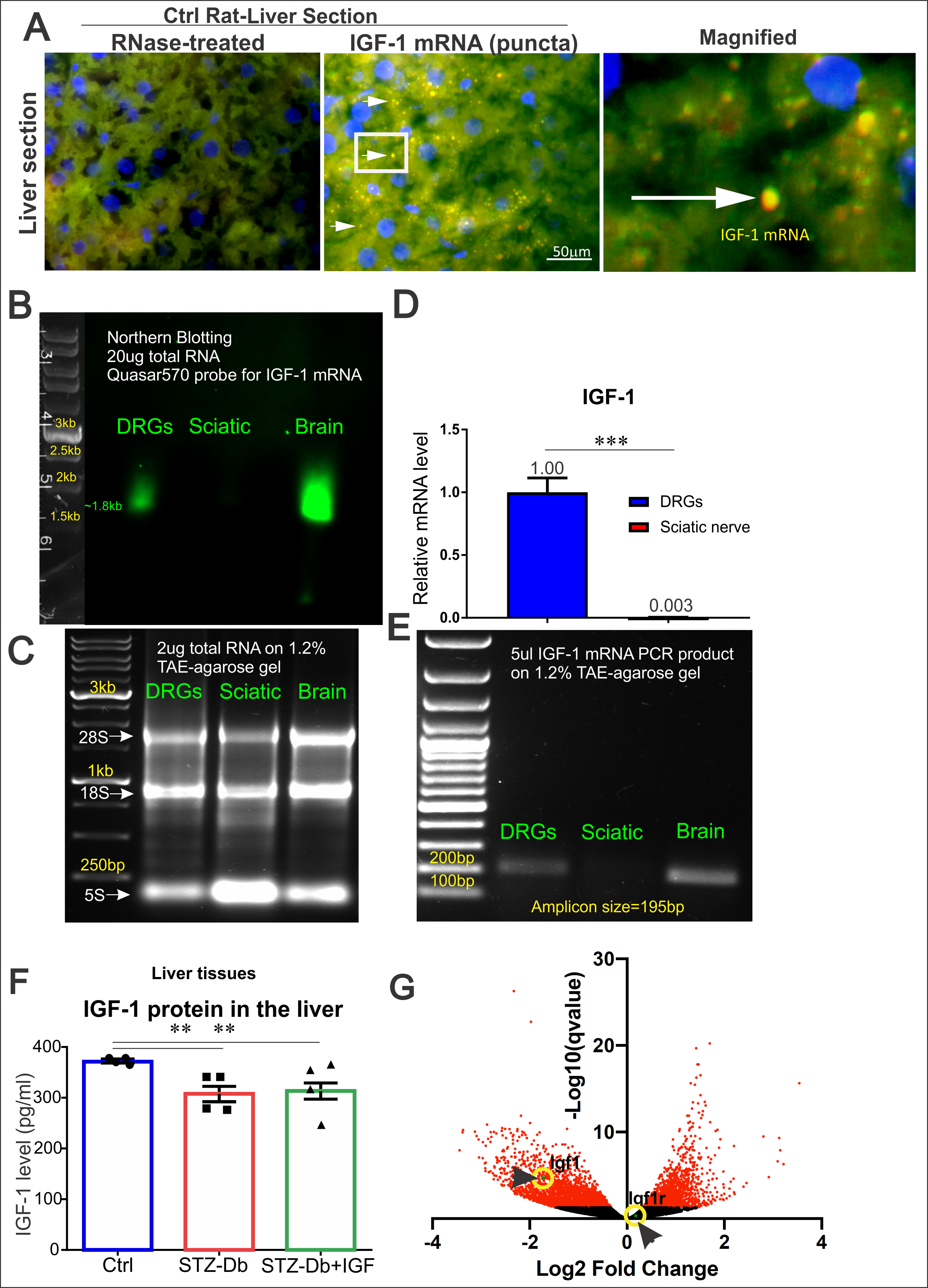

Supplement: Supplementary file 2 — Supplementary file2 Supplemental Fig. 1: Endogenous IGF-1 was detectable in the liver, reduced in diabetic rodents, and expressed at a higher level in brain cortex and DRG tissue vs. sciatic nerve tissue. In (A), liver sections from control rats underwent RNA FISH assay for IGF-1 mRNA detection and localization. The punctate pattern of IGF-1 staining represents its mRNA. Images are magnified for clarification. As a control, tissue sections and cells were exposed to RNase enzyme before hybridizing with IGF-1 probes. In (B-D), DRG, sciatic nerve and brain cortex tissues were obtained from control rats and underwent (B) Northern blotting or (D) Real-Time PCR assay. In (C), 2 µg RNA was run on 1.2% TAE-agarose gel for quality control in (B) and normalization purposes in (D). IGF-1 fluorescent probes were used for hybridization in (B). In (E), PCR products from (D) were run on 1.2% TAE-agarose gel to compare the band intensities. In (F), liver tissues from control (Ctrl), hIGF-1-treated (STZ-Db + hIGF-1) and untreated STZ-diabetic (STZ-Db) rats were homogenized and underwent ELISA for IGF-1 detection. In (G), Volcano plot of upregulated and downregulated genes in the transcriptome of DRG of mice on Western diet vs. normal chow is illustrated (n = 2 biological replicates, biological replicates are 9 DRG from 3 mice). In red significant hits, q value < 0.05, green dots in circle represent igf1 and igf1r as indicated. Data (A-F) are mean ± SEM of N = 3–4; * = p < 0.05 or ** = p < 0.01 or *** = p < 0.001; analyzed by Student’s t test or one-way ANOVA with Tukey’s post hoc test. (TIF 29807 KB) [file 18_2022_4201_MOESM2_ESM.tif]

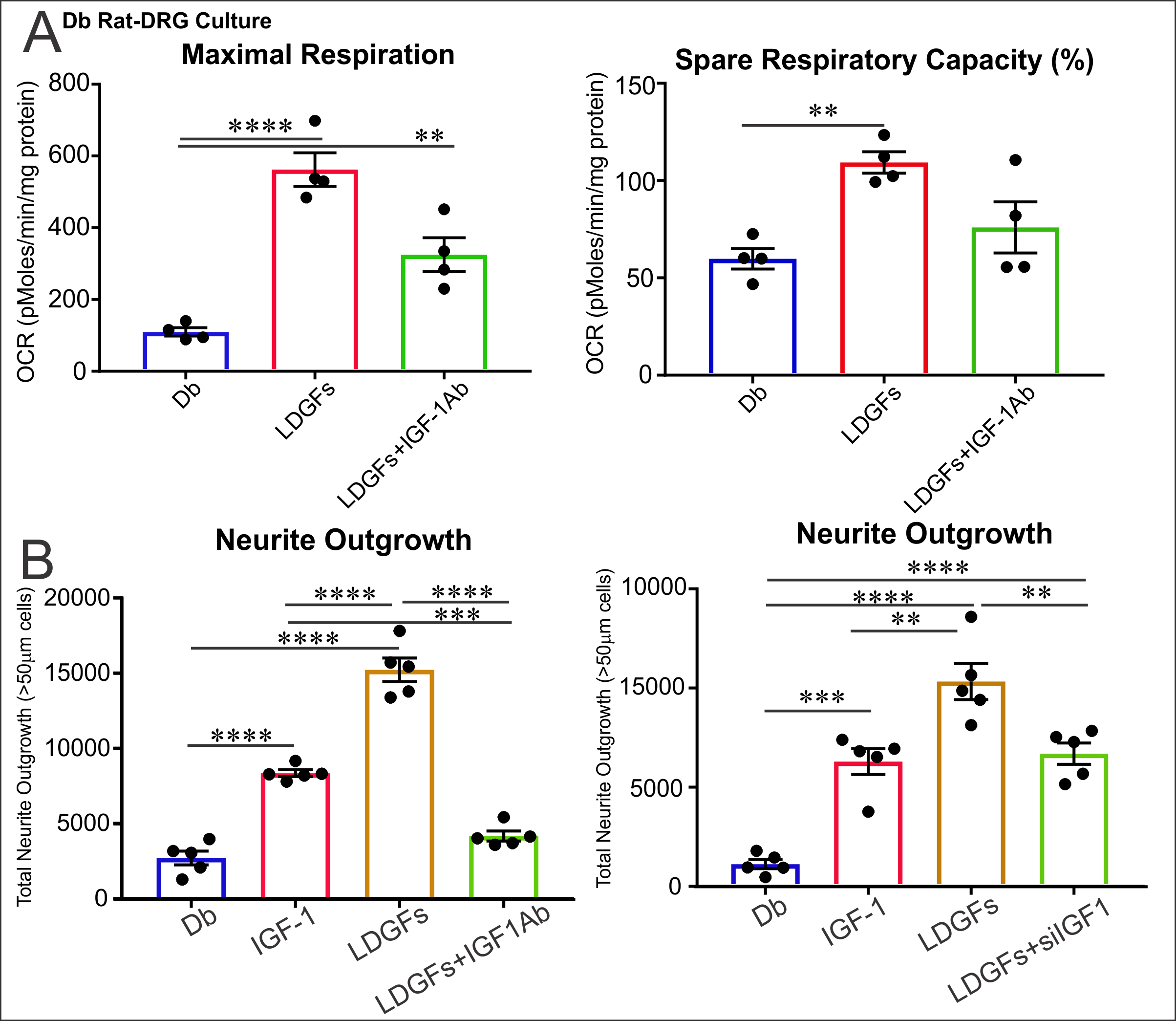

Supplement: Supplementary file 3 — Supplementary file3 Supplemental Fig. 2: IGF-1 neutralizing antibody modulated the neurotrophic factor-dependent elevation of mitochondrial respiration and neurite outgrowth. DRG tissues from STZ-diabetic (Db) rats were cultured in the presence of low-dose growth factors (LDGFs: NGF, GDNF and NT-3) and/or IGF-1 neutralizing antibody. In (A), OCR data were normalized to total protein in mg in each treatment group. In (B), sensory neurons larger than 50 microns in diameter (traditionally classified as large diameter DRG neurons) were selected for analysis since they showed stronger phenotype. Data are mean ± SEM of N = 3–5; * = p < 0.05 or ** = p < 0.01 or *** = P < 0.001 or **** = P < 0.0001; analyzed by one-way ANOVA with Dunnett’s or Tukey’s post hoc test. (TIF 14747 KB) [file 18_2022_4201_MOESM3_ESM.tif]

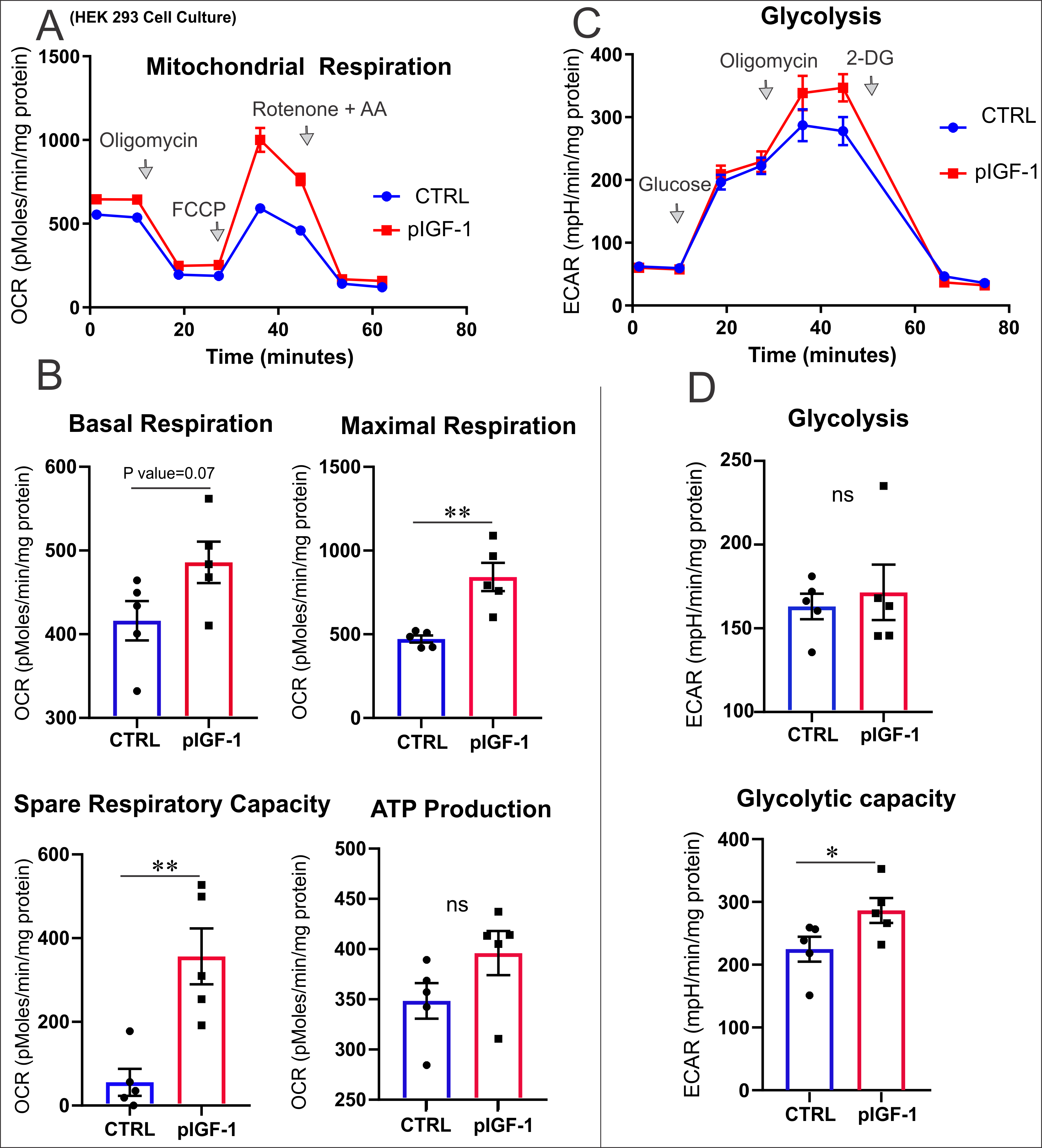

Supplement: Supplementary file 4 — Supplementary file4 Supplemental Fig. 3: IGF-1-overexpressing plasmid enhanced glycolysis and mitochondrial respiration in human HEK293 cell line. HEK293 cells were serum-starved for 6 h, transfected with 2ug hIGF-1 (transcript variant 4)-overexpressing plasmid (pIGF-1) or control GFP plasmid (ctrl). In (A and B), mitochondrial OCR was measured in live cells after 36 h. In (C-F), glycolysis parameters were calculated. Total protein in mg was used to normalize OCR and ECAR data. Data are mean ± SEM of N = 4–5; ** = p < 0.01; analyzed by Student’s t test. (TIF 35562 KB) [file 18_2022_4201_MOESM4_ESM.tif]

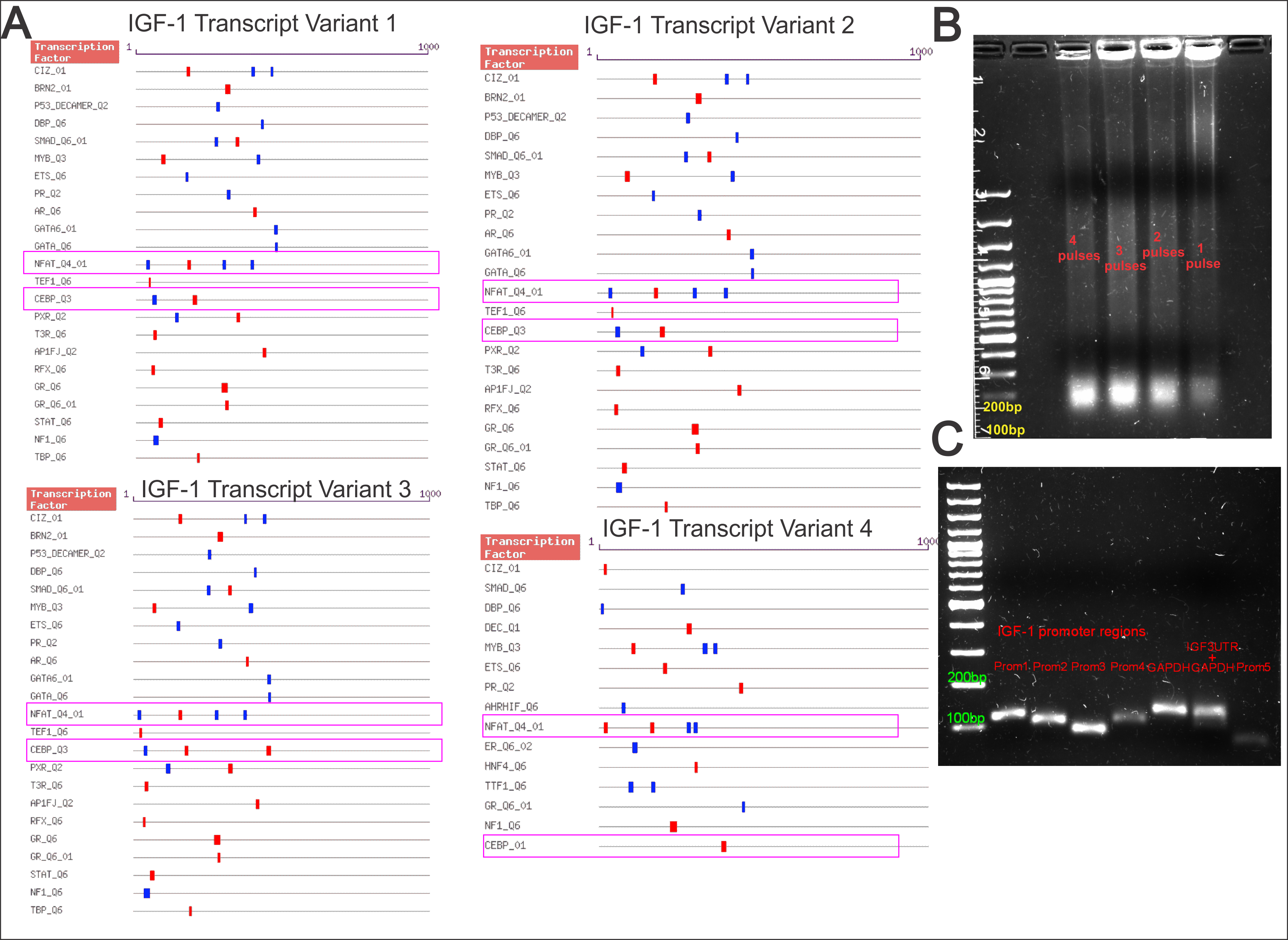

Supplement: Supplementary file 5 — Supplementary file5 Supplemental Fig. 4: Bioinformatic screening of IGF-1 promoter for transcription factor binding and ChIP assay validation. Four transcript variants of rat IGF-1 gene were screened for transcription factor binding on the promoter. The two top inclusive transcription factors, (A) NFAT1 and CEBP-β, were chosen for further experiments. In (B), chromatin fragmentation optimization was performed prior to ChIP experiments. In (C), the validity of PCR products from five promoter regions were tested on an agarose gel. Data are mean ± SEM of N = 5; * = p < 0.05; analyzed by Student’s t test. (TIF 24806 KB) [file 18_2022_4201_MOESM5_ESM.tif]

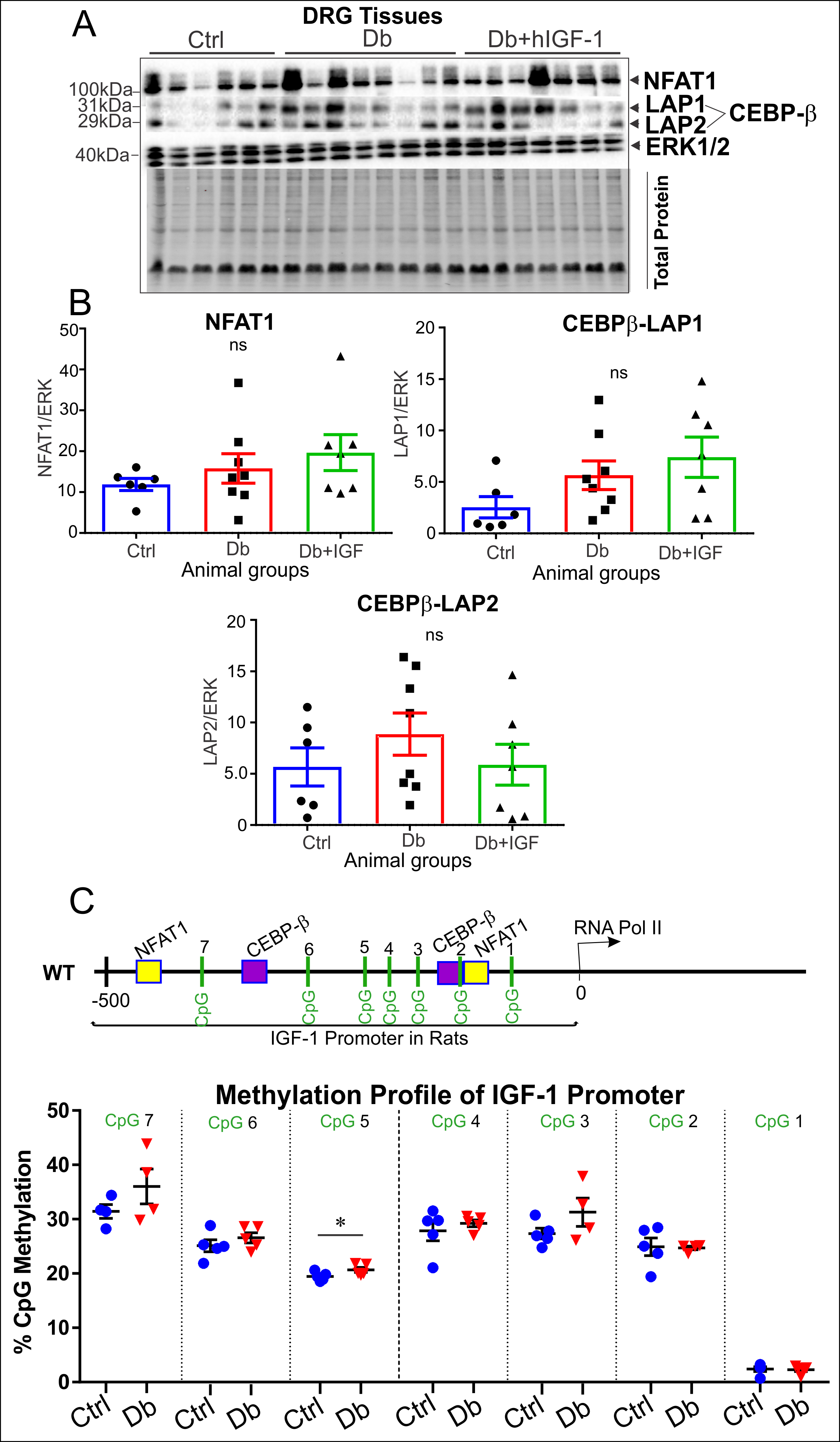

Supplement: Supplementary file 6 — Supplemental Fig. 5: The level of NFAT1 and CEBPβ proteins, and IGF-1 promoter DNA methylation was not significantly changed under diabetic conditions. DRG tissues from control (Ctrl), hIGF-1-treated (Db + hIGF-1) and untreated diabetic (Db) rats were homogenized and underwent (A-B) Western blotting for NFAT1 and CEBP-β (LAP1 and LAP2 isoforms) proteins. Total ERK band intensity was used for normalization. In (C), DNA methylation flanking transcription factor (NFAT1 and CEBPβ) binding sites on IGF-1 promoter was quantified in percent in DRG tissues from control and diabetic rats. Data are mean ± SEM of N = 3–8 animals; analyzed by Student’s t test or one-way ANOVA with Tukey’s post hoc test. (TIF 30799 KB) [file 18_2022_4201_MOESM6_ESM.tif]

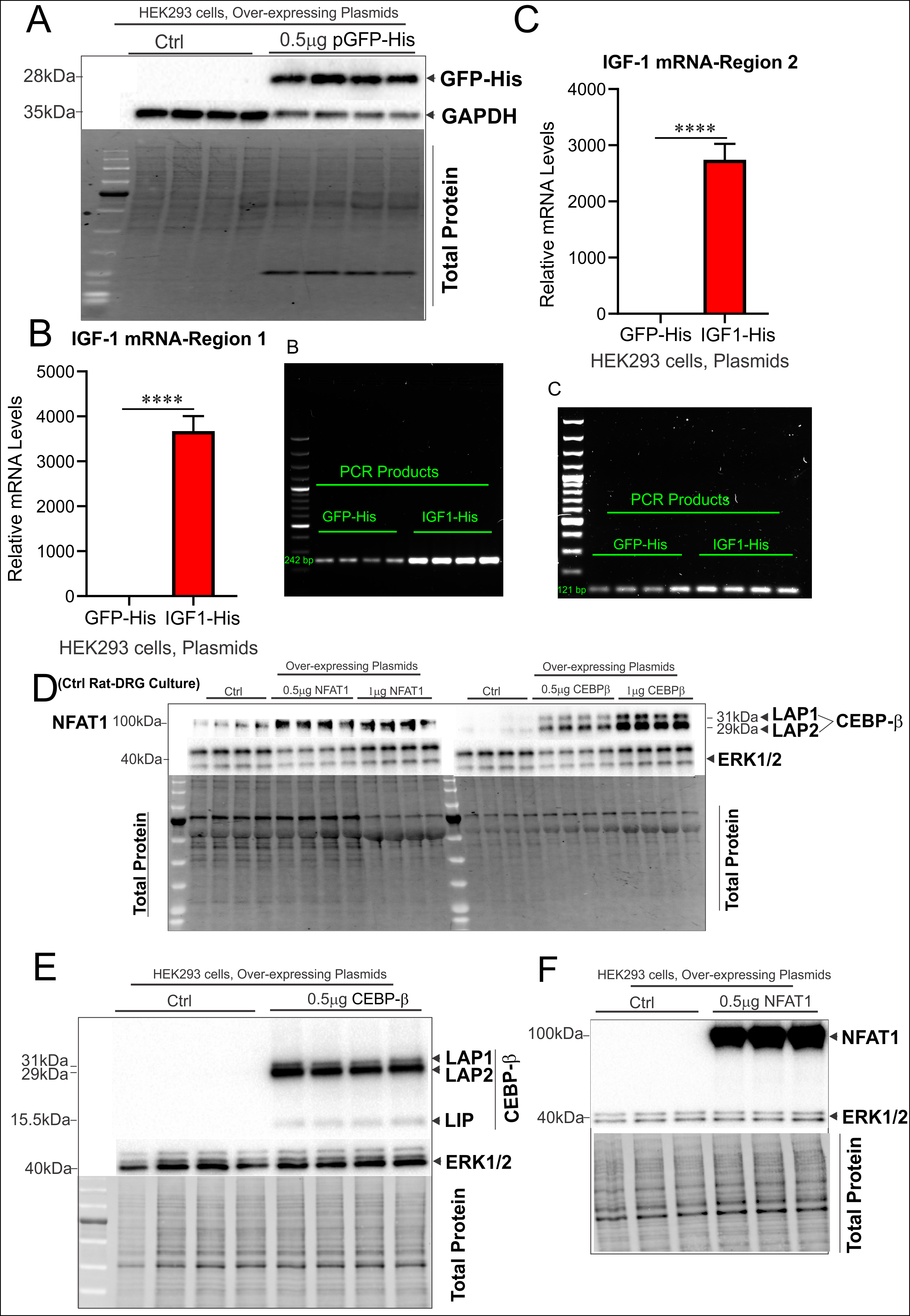

Supplement: Supplementary file 7 — Supplementary file7 Supplemental Fig. 6: Validation of GFP, IGF-1, CEBPβ and NFAT1-overexpressing plasmids in HEK293 cells or DRG neurons. HEK293 cells were cultured and transfected with GFP, IGF-1, CEBPβ or NFAT1 followed by Western blotting or qRT-PCR. In (A), GFP-HisTag was detected using blotting against GFP antibody. In (B and C), two coding regions of IGF1-HisTag plasmid were amplified using qRT-PCR and were run on an agarose gel. CEBPβ and NFAT1 overexpression was confirmed both in (D) DRG culture and (E and F) HEK293 cell line culture using Western blotting. Data are mean ± SEM of N = 4; analyzed by Student’s t test. (TIF 39242 KB) [file 18_2022_4201_MOESM7_ESM.tif]

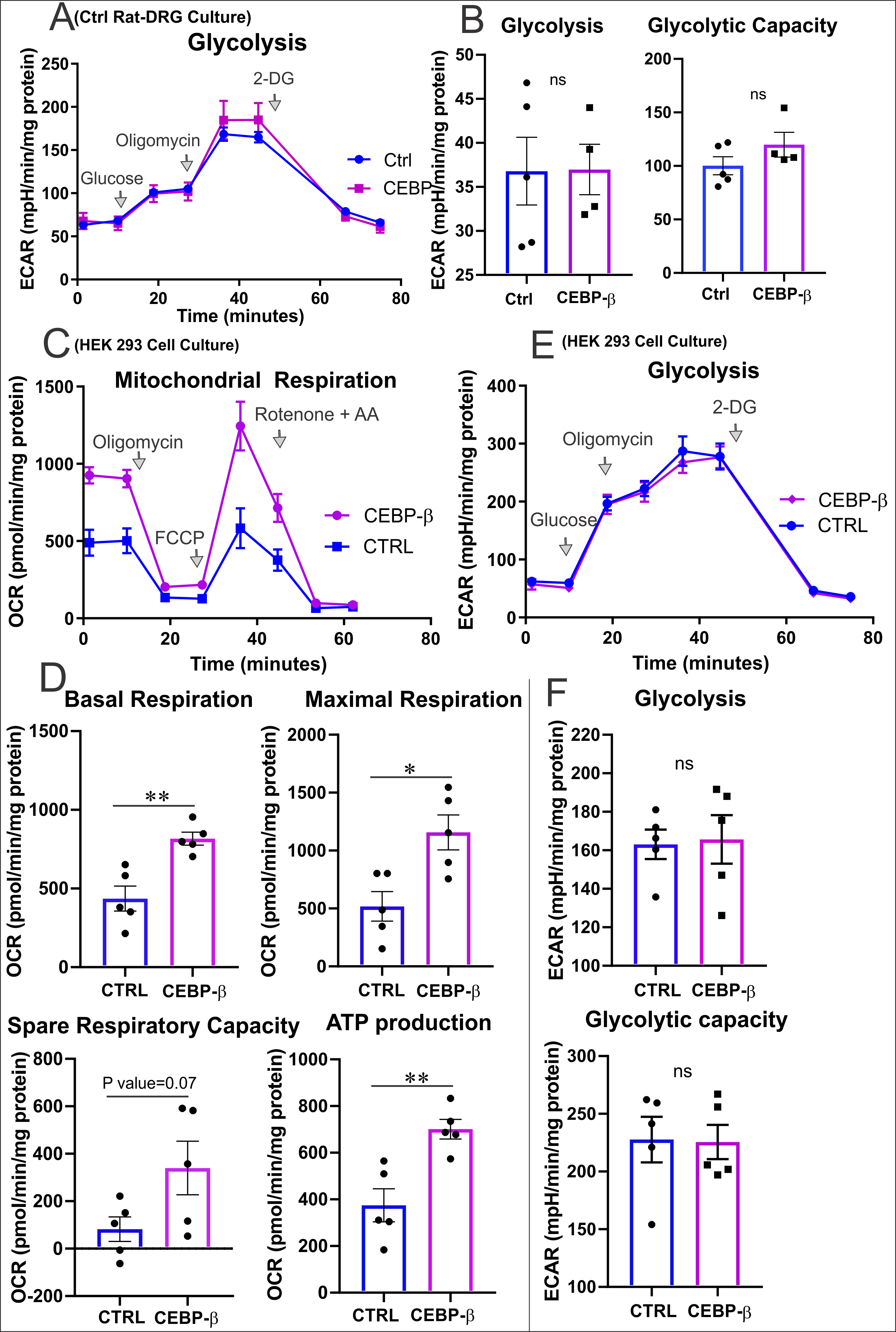

Supplement: Supplementary file 8 — Supplementary file8 Supplemental Fig. 7: CEBPβ-overexpressing plasmid increased mitochondrial respiration but not glycolysis. In (A and B), DRG neurons from control (ctrl) rat were transfected with GFP (ctrl) or 0.5ug CEBPβ-overexpressing plasmids and underwent mitochondrial respiration assay. In (C-F), HEK293 cells were serum-starved for 1 day, transfected with 0.5ug CEBPβ-overexpressing plasmid or control GFP plasmid (ctrl). In (C and D), mitochondrial OCR was measured in live cells after 36 h. In (E and F), glycolysis parameters were calculated. Total protein in mg was used to normalize OCR and ECAR data. Data are mean ± SEM of N = 4–5; * = p < 0.05 or ** = p < 0.01; analyzed by Student’s t test. (TIF 36158 KB) [file 18_2022_4201_MOESM8_ESM.tif]

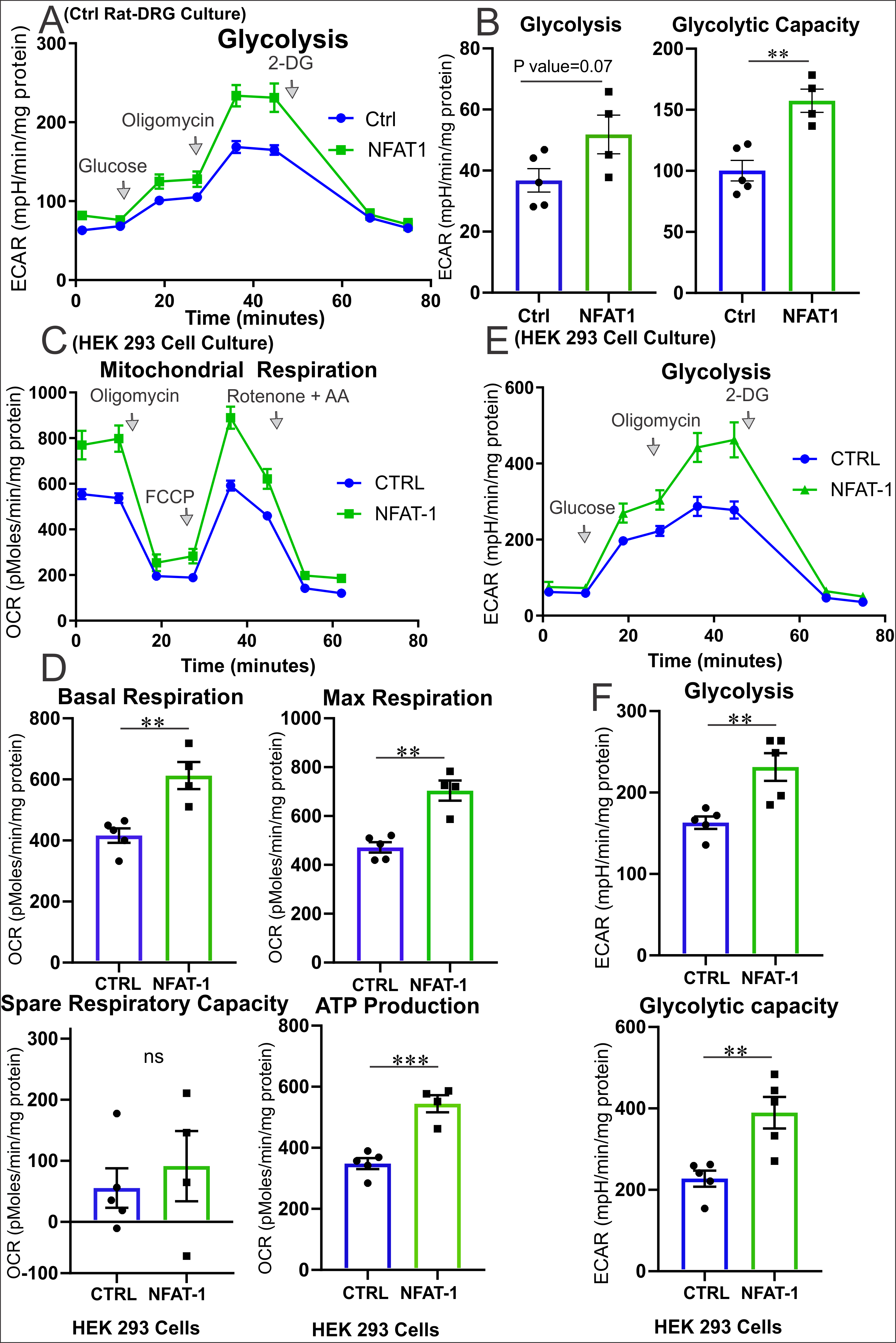

Supplement: Supplementary file 9 — Supplementary file9 Supplemental Fig. 8: NFAT1-overexpressing plasmid upregulated mitochondrial respiration and glycolysis. In (A and B), DRG neurons from control (ctrl) rat were transfected with GFP (ctrl) or 0.5ug NFAT1-overexpressing plasmids and underwent glycolysis assay. In (C-F), HEK293 cells were serum-starved for 1 day, transfected with 0.5ug NFAT1-overexpressing plasmid or control GFP plasmid (ctrl). In (C and D), mitochondrial OCR was measured in live cells after 36 h. In (E and F), glycolysis parameters were calculated. Total protein in mg was used to normalize OCR and ECAR data. Data are mean ± SEM of N = 4–5; ** = p < 0.01 or *** = p < 0.001; analyzed by Student’s t test. (TIF 37811 KB) [file 18_2022_4201_MOESM9_ESM.tif]

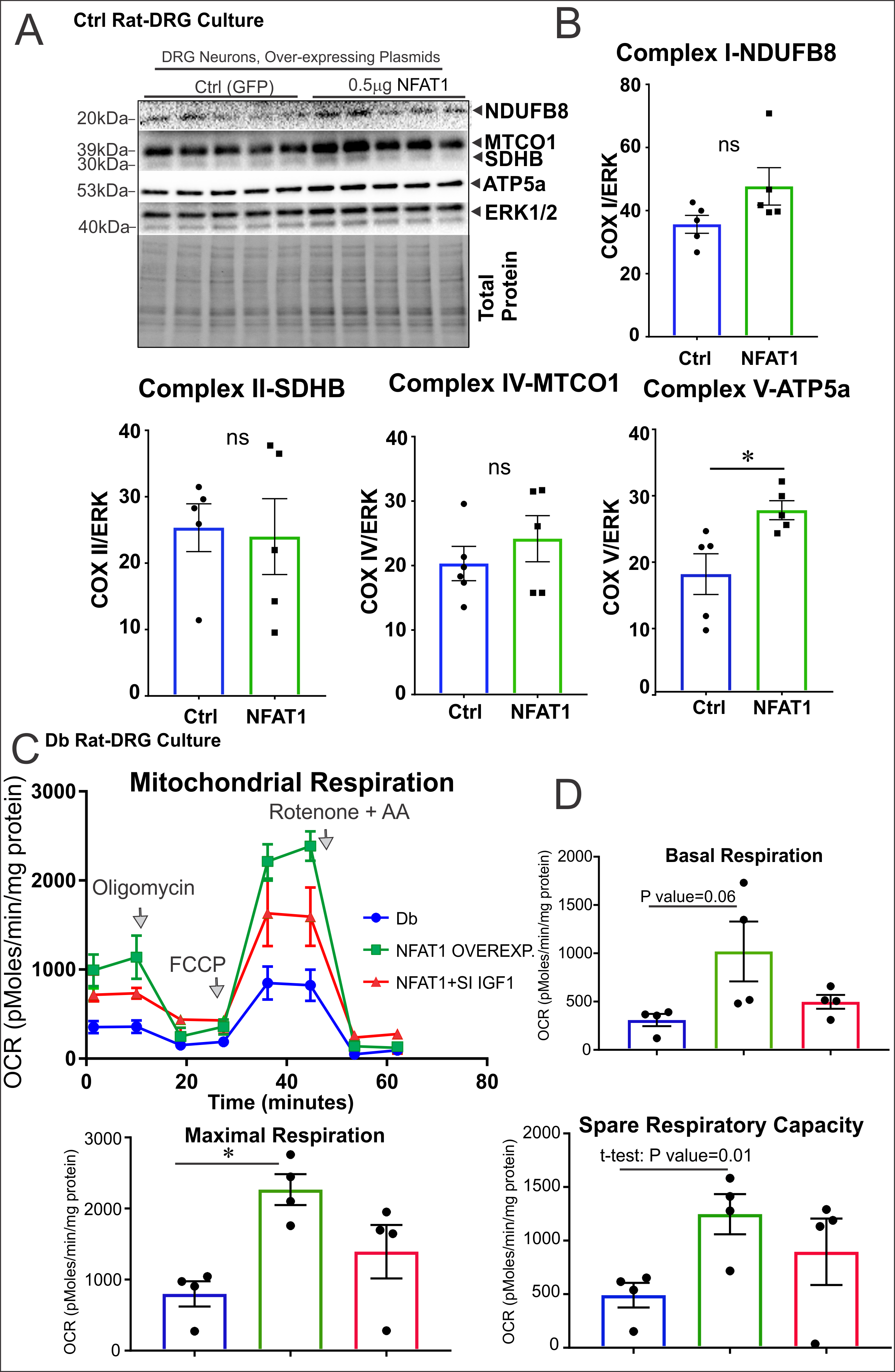

Supplement: Supplementary file 10 — Supplementary file10 Supplemental Fig. 9: NFAT1 overexpression increased ATP5a level and mitochondrial respiration in DRGs, and IGF-1 knock-down modulated the elevated OCR level. DRG neurons from control (ctrl) or STZ-diabetic (Db) rats were cultured, transfected with NFAT1 or GFP plasmids or siIGF1–LNP. In (A and B), DRG tissues from control rats were subjected to Western blotting for mitochondrial ETS proteins. In (C and D), DRGs from Db rats were cultured, transfected with either GFP plasmid or NFAT1 plasmid and/or siIGF–LNP for 36 h and subjected to mitochondrial respiration analysis. Total protein in mg was used to normalize OCR data. Data are mean ± SEM of N = 4–5; * = p < 0.05; analyzed by Student’s t test or one-way ANOVA with Tukey’s post hoc test. (TIF 26670 KB) [file 18_2022_4201_MOESM10_ESM.tif]
